# Computational design and investigation of the monomeric spike SARS-CoV-2-ferritin nanocage vaccine stability and interactions

**DOI:** 10.3389/fmolb.2024.1403635

**Published:** 2024-06-12

**Authors:** Farnaz Garmeh Motlagh, Maryam Azimzadeh Irani, Seyedeh Zeinab Masoomi Nomandan, Mohammad Assadizadeh

**Affiliations:** Faculty of Life Sciences and Biotechnology, Shahid Beheshti University, Tehran, Iran

**Keywords:** nanoparticle vaccine, ferritin, SARS-CoV-2, monomeric spike, molecular docking, molecular dynamics simulation

## Abstract

Since the Severe Acute Respiratory Syndrome Coronavirus 2 (SARS-CoV-2) outbreak, several solutions have been proposed to manage the disease. The most viable option for controlling this virus is to produce effective vaccines. Most of the current SARS-CoV-2 vaccines have focused on the infusion spike protein. Spike exists as a trimer and plays a vital role in infecting host cells by binding to the Angiotensin-Converting Enzyme 2 (ACE2) receptor through its Receptor Binding Domain (RBD). Ferritin protein, a naturally occurring iron-storage protein, has gained attention for vaccine production due to its self-assembling property, non-toxic nature, and biocompatibility. Ferritin nanocages have recently been employed in the development of a SARS-CoV-2 vaccination eliciting not only long-term protective memory cells but also a sustained antibody response. In this study, a combination of *in silico* investigations including molecular docking, molecular dynamics simulations, and immune simulations were carried out to computationally model the monomeric spike protein on the ferritin nanocage as well as to evaluate its stability and interactions for the first time. The structural dynamics of the modeled complex demonstrated noticeable stability. In particular, the Receptor Binding Domain (RBD) and ferritin within the monomeric spike-ferritin complex illustrated significant stability. The lack of alterations in the secondary structure further supported the overall steadiness of the complex. The decline in the distance between ferritin and spike suggests a strong interaction over time. The cross-correlation matrices revealed that the monomeric spike and ferritin move towards each other supporting the stable interaction between spike and ferritin. Further, the orientation of monomeric spike protein within the ferritin unit facilitated the exposure of critical epitopes, specifically upward active Receptor Binding Domain (RBD), enabling effective interactions with the ACE2 receptor. The immune simulations of the model indicated high-level stimulations of both cellular and humoral immunity in the human body. It was also found that the employed model is effective regardless of the mutated spikes in different variants. These findings shed light on the current status of the SARS-CoV-2-ferritin nanoparticle vaccines and could be used as a framework for other similar vaccine designs.

## Graphical Abstract

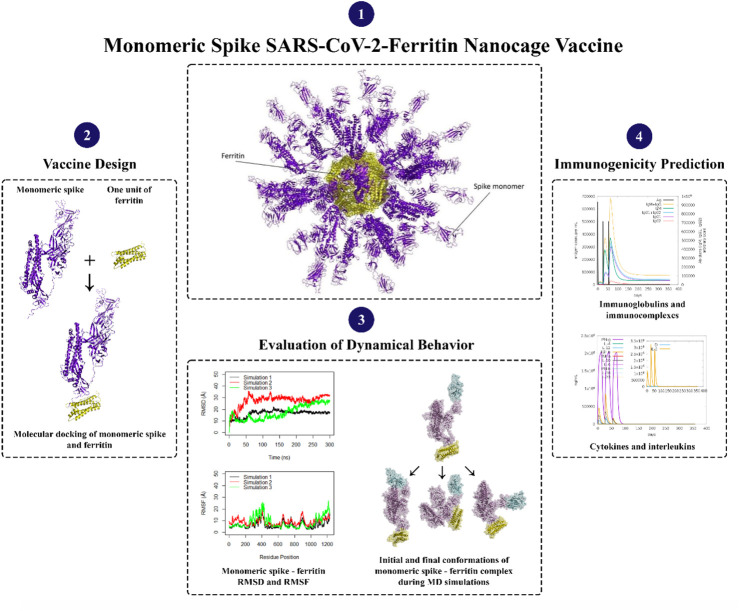

## Introduction

**FIGURE 1:**
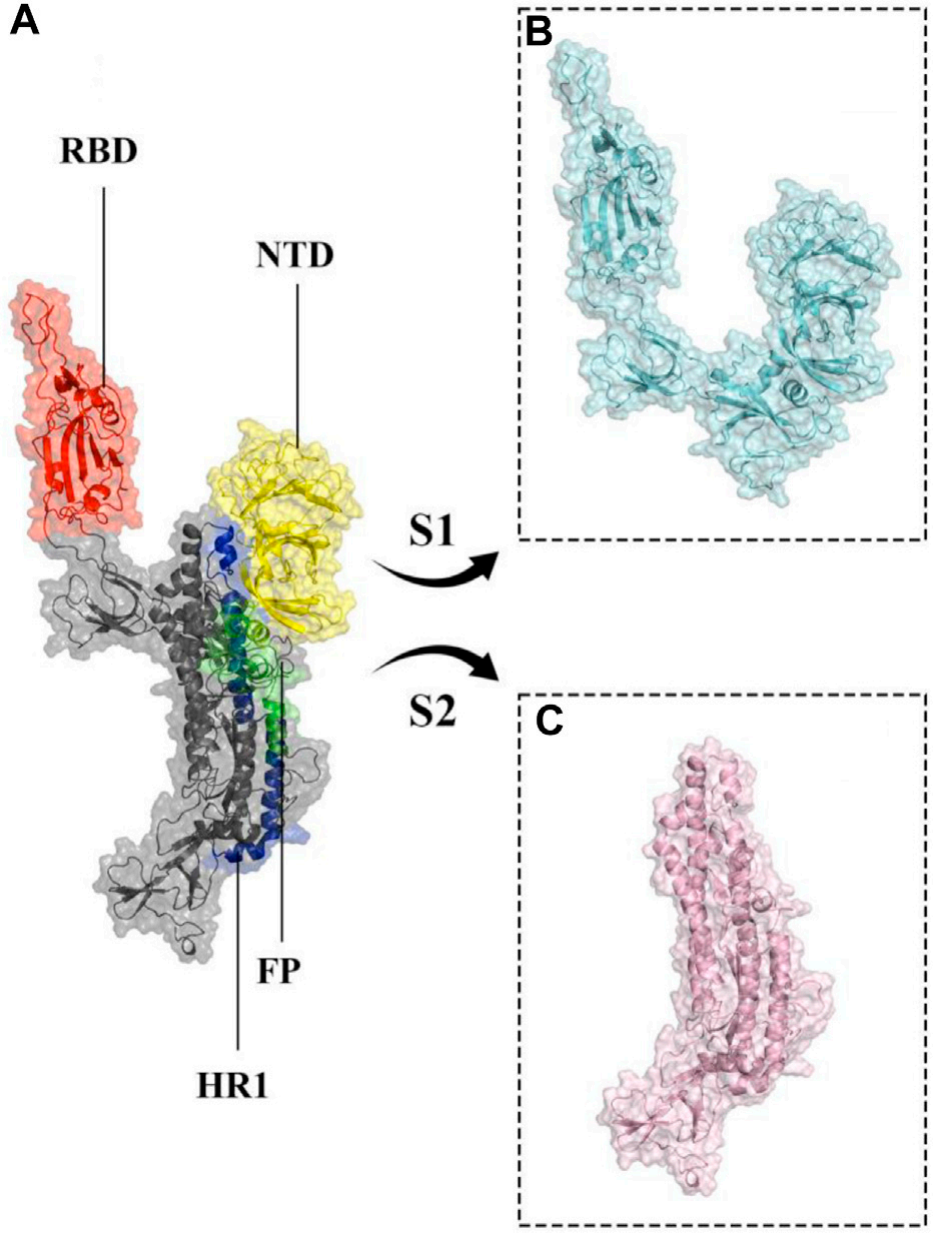
Structural representation of the monomeric spike SARS-CoV-2 ectodomain. **(A)** monomeric spike in gray cartoons and transparent surfaces. Its different parts, including Receptor Binding Domain (RBD), N-terminal domain (NTD), Fusion Peptide (FP), and Heptad Repeat 1 (HR1) are shown in red, yellow, green, and blue cartoon and transparent surfaces respectively. **(B, C)** subunits S1 and S2 in cyan and light pink cartoon and transparent surfaces, respectively.

More than 773 million people have been affected, and over six million have died due to the global coronavirus disease 2019 (COVID-19) pandemic caused by severe acute respiratory syndrome coronavirus 2 (SARS-CoV-2) (Acuti Martellucci et al., 2020; World Health Organization, 2024; Wu et al., 2020). SARS-CoV-2 is a single-stranded RNA virus with a positive sense and large RNA genome, four structural proteins, 16 nonstructural proteins, and 9–11 accessory proteins (Alexandersen et al., 2020; Malik, 2020). The four structural proteins are spike, envelope, membrane, and nucleocapsid proteins, with the spike glycoprotein (S protein) being particularly important as it is a prominent coronavirus vaccine target (Andersen et al., 2020; Choi et al., 2021; Du et al., 2009; Hu et al., 2021; Walls et al., 2020; Wrapp et al., 2020). SARS-CoV-2 enters cells by binding to the host cellular receptor Angiotensin-Converting Enzyme 2 (ACE2) via its spike protein (Song et al., 2018; Zhao et al., 2020; Piplani et al., 2021; Jackson et al., 2022), and typically causes a lower respiratory tract infection, which can progress to severe acute respiratory syndrome and potentially multiple organ failure (Ganji et al., 2020; Tu et al., 2020; Tao et al., 2021; Yang et al., 2021). The S protein monomer, initially produced as a single polypeptide chain, is made up of a fusion peptide, two heptad repeats, an intracellular domain, an N-terminal domain, two subdomains, and a transmembrane region (Figure 1). After translation it is cleaved into S1 and S2 subunits (Li et al., 2005; Gui et al., 2017). A 25 KDa Receptor Binding Domain (RBD; residue range 334–527) within S1 interacts with the ACE2 receptor (Hoffmann et al., 2020; Walls et al., 2020). As a result, the S protein dictates SARS-COV-2’s infectivity and transmissibility (Du et al., 2009; Yang et al., 2020).

Developing a safe and effective vaccine for SARS-CoV-2 and plausible emerging variants is a top priority for public health, and long-term pandemic control will necessitate one or more effective vaccines that can be widely distributed worldwide (Chen W.-H. et al., 2020; Haiou et al., 2022). Protein subunit vaccines and genetically encoded nucleic acid vaccines are the most effective for the prevention and treatment of SARS-CoV-2 disease (Amanat and Krammer, 2020). Protein nanoparticles which have been vastly used in therapeutic purposes possess a high surface area to volume ratio, enhancing their drug-holding capacity, the solubility of the drug, and their bioavailability (Bhushan et al., 2014).

**FIGURE 2:**
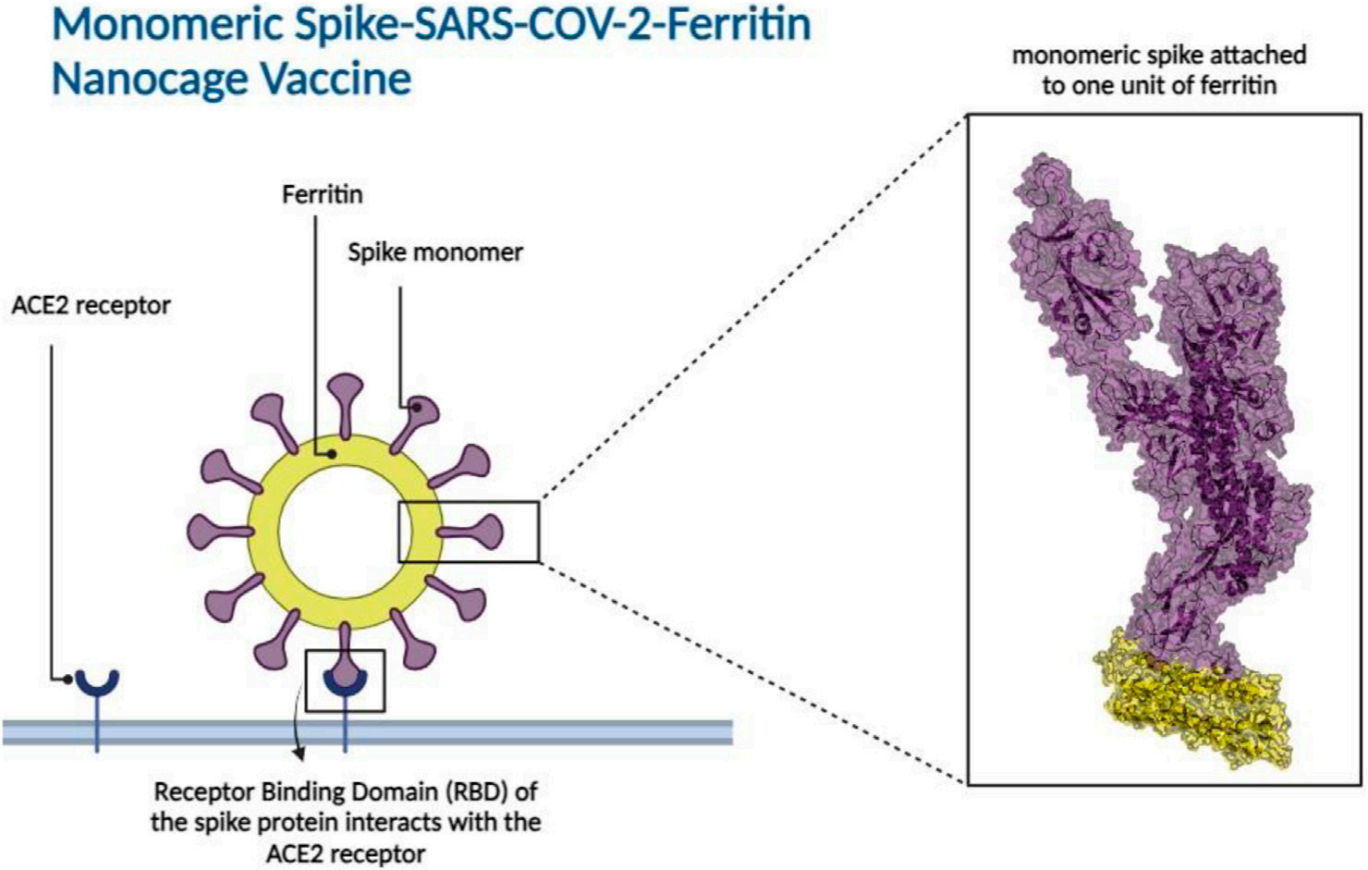
Monomeric spike SARS-CoV-2 - Ferritin nanocage vaccine design. The interaction between the Receptor Binding Domain (RBD) of the monomeric spike protein and the Angiotensin-Converting Enzyme 2 (ACE2) receptor found in human cells is illustrated in the left panel. The box represents the 3D structural presentation of one monomeric spike attached to a unit of ferritin. Spike and ferritin are presented with purple and yellow cartoon and transparent surfaces, respectively.

Ferritin, a family of protein cages that plays a crucial function in iron storage, is highly evolutionarily ubiquitous (Andrews et al., 1992; Uchida et al., 2010; Jutz et al., 2015). Ferritin is a 24-meric cage with octahedral symmetry and a protein nanoparticle (Lawson et al., 1991; Zhang and Orner, 2011; Chakraborti and Chakrabarti, 2019). Ferritin’s surfaces are open to various types of changes, which is one of the reasons behind its utility for biological applications (Jin et al., 2019), including vaccine development. Ferritin nanocages have been utilized to display antigens from various pathogens (Bachmayer et al., 1976; Zhang and Orner, 2011; Kanekiyo et al., 2013; Stone et al., 2013; Kanekiyo et al., 2015; He et al., 2016; Chattopadhyay et al., 2017; Qi et al., 2018; Jin et al., 2019; Kamp et al., 2020; Swanson et al., 2020; Yao et al., 2020; Wuertz et al., 2021), all of which elicit a robust immune response. Nanoparticles in vaccine formulations are predicted to improve antigen stability and immunogenicity, as well as targeted distribution and gradual release (Oyewumi et al., 2010; Carmen et al., 2021; Gordon et al., 2022). Various types of nanoparticles have been utilized in vaccine development (Glück et al., 2004; Borges et al., 2008; Champion et al., 2009; Nochi et al., 2010; Zhu et al., 2010; Wang et al., 2011; Zhao et al., 2012; Bhushan et al., 2014; Zhao et al., 2014; Sliepen et al., 2015; Edwardson and Hilvert, 2019; Swanson et al., 2020). Synthetic vaccines are not only safer than attenuated or inactivated microorganisms, but they also allow for custom vaccine development (Liu et al., 2014). To produce the SARS-CoV-2 vaccine, the spike protein is displayed as an immune system stimulator on ferritin units. The ferritin-base vaccines elicited a long-lasting antibody response and a remarkable long-term memory demonstrating the efficacy of ferritin nanoparticles in preserving antibody response (Kalathiya et al., 2021; Powell et al., 2021; Wang et al., 2021). Recent research indicated that when the RBD and ferritin nanoparticles were stitched together, the resulting synthetic chimeras displayed the RBD effectively and elicited moderate to high-effect immune responses in experimental animal models (Kanekiyo et al., 2013; Qi et al., 2018; Petersen et al., 2020; Yao et al., 2020; Powell et al., 2021). *In silico* studies of linker and glycosylation design to improve nanoparticle vaccine constructs have been recently investigated (Kalathiya et al., 2021; Masoomi Nomandan et al., 2022). Specifically, the *in silico* ferritin-SARS-CoV-2 glyco-RBD nanoparticle vaccine has demonstrated significant efficacy against the virus (Masoomi Nomandan et al., 2022). These studies predominantly focused on the RBD of the spike protein (Amanat et al., 2020; Chen et al., 2020; Mulligan et al., 2020). However, experimental evidence has suggested that the potential stability of a full-length spike attached to ferritin seems to be higher than that of the RBD attached to ferritin (Powell et al., 2021). In this study, the attachment of monomeric spike to ferritin (Figure 2) was explored. A combination of molecular docking, molecular dynamics simulations, and immune simulations were carried out to assess the resulting designed vaccine in terms of structural stability and interactions.

## Computational methods

### 3D structures of monomeric spike, ferritin and lectin

The 3D structure of the SARS-CoV-2 spike ectodomain in an open state, represented by PDB ID 6VYB (Walls et al., 2020), was chosen for the monomeric spike protein. This structure was obtained through electron microscopy, with a resolution of 3.20 Å, and was released on 2020–03-11. The selection of 6VYB was based on its representation of the spike protein in a conformation relevant to its interaction with host cells, which is crucial for eliciting an immune response against the virus antigen. The crystal structure of human L-ferritin, represented by PDB ID 2FG8 (Wang et al., 2006), was selected for the ferritin protein. This structure was determined using X-ray diffraction, with a resolution of 2.50 Å, and was released on 2006–07-04. PDB ID 2FG8 was chosen for its well-defined atomic details, which is crucial for accurate structural analysis of ferritin in the designed vaccine. The lectin protein structure was obtained from PDB ID 5B1W (Nagae et al., 2016). This structure was determined using X-ray diffraction, with a resolution of 3.05 Å, and was released on 2016–05-11.

### Molecular docking of monomeric spike and ferritin

The selected structure of the monomeric spike was used to dock to one unit of ferritin with the HADDOCK server (van Zundert et al., 2016), where the HADDOCK server performed rigid body energy minimization in the initial step of the docking process. Residues at the C-terminal of monomeric spike (residues: 1,136–1,147) were chosen as a binding interface area according to previous studies (Powell et al., 2021; Wuertz et al., 2021; Gordon et al., 2022) and several docking scores (Supplementary Figure S1). One exposed loop on the ferritin structure (residues:73–91) was selected as the most plausible binding site (Masoomi Nomandan et al., 2022), which was confirmed by comparing the energetic terms and clustering of the resulting docked poses. The docked pose with the minimum HADDOCK score and lowest RMSD values was considered for model development. The poses were validated via further molecular docking and processed by the ClusPro web server (Supplementary Table S1) (Kozakov et al., 2017). Details outlining the atomistic interactions of the modeled construct are provided (Supplementary Table S2; Supplementary Figure S2). Biomolecular visualization of the complex was carried out by PyMOL (LLC, 2015).

### Molecular docking of monomeric spike-ferritin complex and lectin

Lectins are a unique class of proteins or glycoproteins known for their ability to selectively recognize carbohydrate structures and form reversible linkages upon interacting with glycoconjugate complexes (Raposo et al., 2021). The molecular docking analysis examined the interaction between the monomeric spike-ferritin complex and lectin molecules, aiming to explore the potential interference of lectin with vaccine efficacy. HADDOCK server (van Zundert et al., 2016) was employed to dock full-length human lectin and monomeric spike-ferritin nanoparticle vaccine together (Supplementary Figure S3). It was observed that seven residues involved in the ferritin-lectin binding interface were also found to participate in the ferritin-monomeric spike interface. However, the most stable cluster exhibited a Root Mean Squared Deviation (RMSD) value of 4.7±0.0 Å and a HADDOCK score of 163.2±41.3. This suggests that the connection between lectin and the constructed vaccine is weak and unstable, and might not significantly impact the vaccine efficacy. These observations were further validated through ferritin and lectin docking (Supplementary Figure S4). The most stable cluster displayed an RMSD value of 20.1±0.2 Å and a HADDOCK score of 188.3±23.4. Based on these results, it is unlikely that ferritin or the monomeric spike would be bound to lectin proteins in a manner that would significantly interfere with their intended roles in the vaccine’s function.

### Molecular dynamics simulations and analyses

The dynamic behavior of the monomeric spike SARS-CoV-2-ferritin complex was investigated through molecular dynamics simulations using GROMACS 2021.4 software (Hess et al., 2008). OPLS-AA/M force field (Jorgensen et al., 1996) was employed to generate parameters and topology files. The system was solvated in a cubic box of water molecules with a volume of 3,140.21 nm³, ensuring a 10 Å boundary around the proteins. TIP3P (Jorgensen et al., 1983) was used to simulate water molecules and counter ions were added to neutralize the system (13 Na + ions). A two-step approach involving 50,000 steps of steepest descent and 50,000 steps of conjugate gradient energy minimization was utilized to relieve the system from steric clashes. In the first step, the steepest descent algorithm was used with a maximum force tolerance of 500 kJ/mol/nm and an energy step size of 0.01 nm. Subsequently, the conjugate gradient algorithm was applied with a maximum force tolerance of 100 kJ/mol/nm and an energy step size of 0.01 nm, until convergence was achieved. The LINCS algorithm (Hess et al., 1997) was employed to constrain the bond lengths. Nonbonded interactions (electrostatic and VDW) were calculated with a cutoff value of 10 Å. The Particle Mesh Ewald method (Darden et al., 1993) with 0.16 nm Fourier grid spacing (Saini et al., 2019) was used for long-range electrostatic interactions. In the initial equilibration phase, the NVT ensemble was utilized to maintain a constant temperature of 310 K throughout a 100 ps simulation with a time step of 1 fs. The system was heated up from 0 K to 310 K over four steps using an annealing method. Following this, the temperature was maintained at 310 K using the V-rescale temperature coupling algorithm, a modified version of the Berendsen thermostat. To ensure structural stability, position restraints were applied to the proteins to prevent large fluctuations. Following the NVT equilibration, the system underwent further equilibration through a 250 ps simulation under an NPT ensemble at 310 K and 1 bar pressure, with a time step of 1 fs. Temperature and pressure coupling were achieved employing V-rescale and Berendsen methods, respectively. Finally, under NPT, with the V-rescale thermostat maintaining 310 K and the Parrinello-Rahman pressure coupling algorithm maintaining 1 bar, three replicates of 300 ns with a time step of 2 fs were conducted.

Bio3D package (Grant et al., 2006) in R was used for calculating the Root Mean Squared Deviation (RMSDs), Root Mean Squared Fluctuations (RMSFs), Principal Component Analysis (PCA), and Cross-Correlation of Cα atoms (DCCMs) for each replicate simulation during 300 ns of simulation time. Distances between centers of masses of spike and ferritin were calculated using VMD (Visual Molecular Dynamics) software (Humphrey et al., 1996). Secondary structure timeline analyses were calculated over 300 ns of simulation time with VMD STRIDE plugin. VMD was also utilized for calculating Solvent Accessible Surface Area (SASA). The visualization of structures was performed using PyMOL (LLC, 2015) and Figure 2 was created using BioRender.com.

### Indicator for immune response simulation

The immunological simulation of the monomeric spike-ferritin vaccine was performed using the C-IMMSIM server (Rapin et al., 2010; Castiglione et al., 2021) to describe the immune response profile and immunogenicity of the antigenic peptide. The experiment was carried out by adding FASTA sequences of vaccine constructions into the simulation, which lasted approximately 12 months (a time step is about 8 h). The random seed and simulation volume was set to 12,345 and 50. Three *in silico* injections with no LPS were administered at time steps 1, 84, and 168, with a minimum time interval of 30 days between two injections. Additionally, to ensure the reliability of the simulation results, two additional simulations were carried out, with the random seed set to different numbers: one with the seed 3,452 and the other with 15,342 (Supplementary Figures S5, 6).

### Predicting T cell epitopes on the monomeric spike protein of the designed vaccine

The peptides from monomeric spike proteins of the designed vaccine that are naturally processed by Major Histocompatibility complex (MHC) class I molecules were predicted using the Immune Epitope Database (IEDB) (Vita et al., 2019). This analysis employed the IEDB MHC prediction tool, MHC-NP (Giguère et al., 2013), which utilizes machine learning algorithms to predict peptides with potential binding affinity to MHC class I molecules. The ectodomain sequence of the monomeric spike protein of SARS-CoV-2 was utilized as the input sequence for the analysis. To ensure relevance to human immune responses, the chosen MHC source species was human. Additionally, to ensure comprehensive coverage of potential T-cell epitopes, five common Human Leukocyte Antigen (HLA)-B variants were selected as the MHC alleles. Peptide lengths ranging from 8 to 11 amino acids (all lengths) were assigned to capture a diverse range of potential epitopes. Finally, 3,620 peptides were identified for each of the examined variants, each demonstrating different affinities for MHC class I binding. Furthermore, MHC-II binding predictions were conducted to identify potential T-cell epitopes, utilizing the NetMHCIIpan EL prediction method (Reynisson et al., 2020). This analysis spanned across the 19 most common HLA-DR alleles, ensuring a broad representation of human genetic diversity. The default peptide length of 15 amino acids was employed for these predictions. This approach aimed to identify epitopes with high binding affinity to MHC-II molecules, thereby enabling investigation into vaccine efficacy across genetic variations in the host immune system.

## Results

### 3D constructs of the monomeric spike within the ferritin nanocage

**FIGURE 3:**
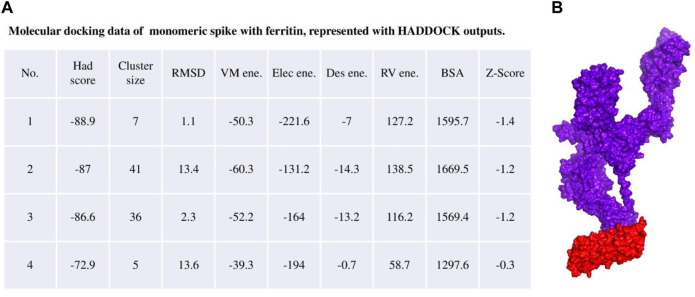
**(A)** Molecular docking result of the monomeric spike with ferritin unit from the HADDOCK server. **(B)** The best binding pose is presented in the right panel in purple surface while the ferritin unit is shown in red surface.

**FIGURE 4:**
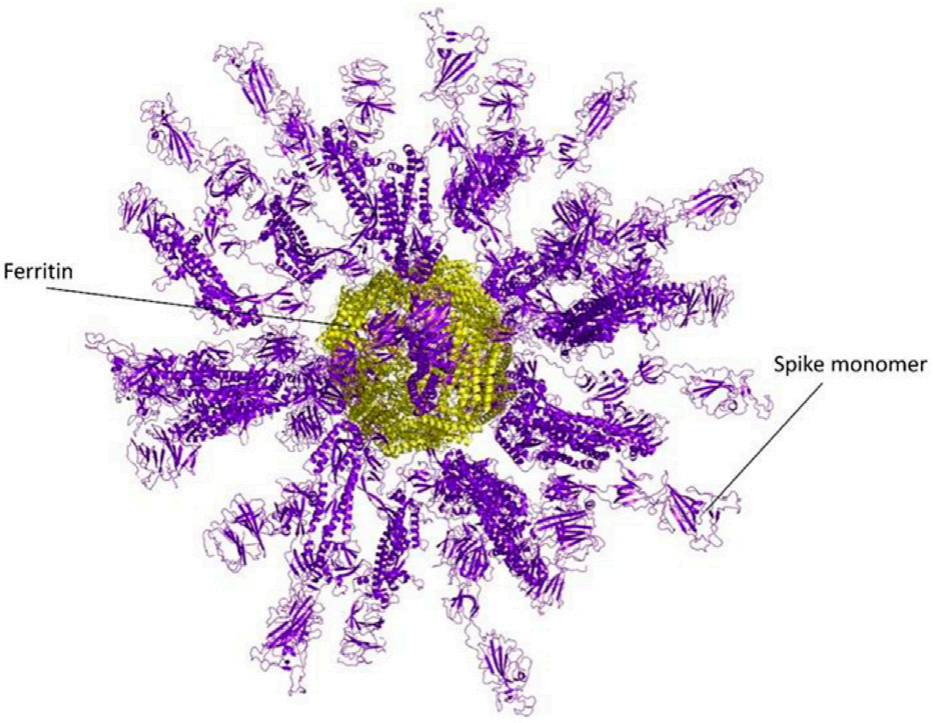
A Full nanocage model of the monomeric spike with ferritin is shown in the picture. Spikes are presented in the purple cartoon, while ferritin units are shown in the yellow cartoon.

The ferritin nanoparticle vaccine was modeled with the monomeric spike antigen by molecular docking. The docking score obtained for the selected binding pose and the RMSD value were −88 and 1.1 (Å), respectively (Figure 3). The ferritin full-cage structure was assembled with 24 ferritin single units and has shown twenty monomeric spikes on ferritin structure, with four ferritin units remaining without adjuvants due to steric hindrance (Figure 4).

### The efficiency of the monomeric spike for inhibition of different SARS-CoV-2 variants

By examining the mutations that occurred in the spike protein of variants of concern, including Alpha, Beta, Gamma, Delta, and Omicron (Supplementary Figure S7), it was noticed that not only do none of the mutations exist in the ferritin binding interface but the center of occurrence of mutations is also far from the binding interface of ferritin in the monomeric spike vaccine model. While the mutations D1118H and H1101D, associated with the Alpha and Beta variants respectively, are in close proximity to the binding interface, it is crucial to note that they are not directly situated within it. The binding interface is specifically defined by residues within the range of 1,136–1,147, where neither D1118H nor H1101D fall within this range.

Thus, the monomeric spike-ferritin vaccine model can still be effective despite the known mutations.

### Structural stability and flexibility of the monomeric spike-ferritin complex

**FIGURE 5:**
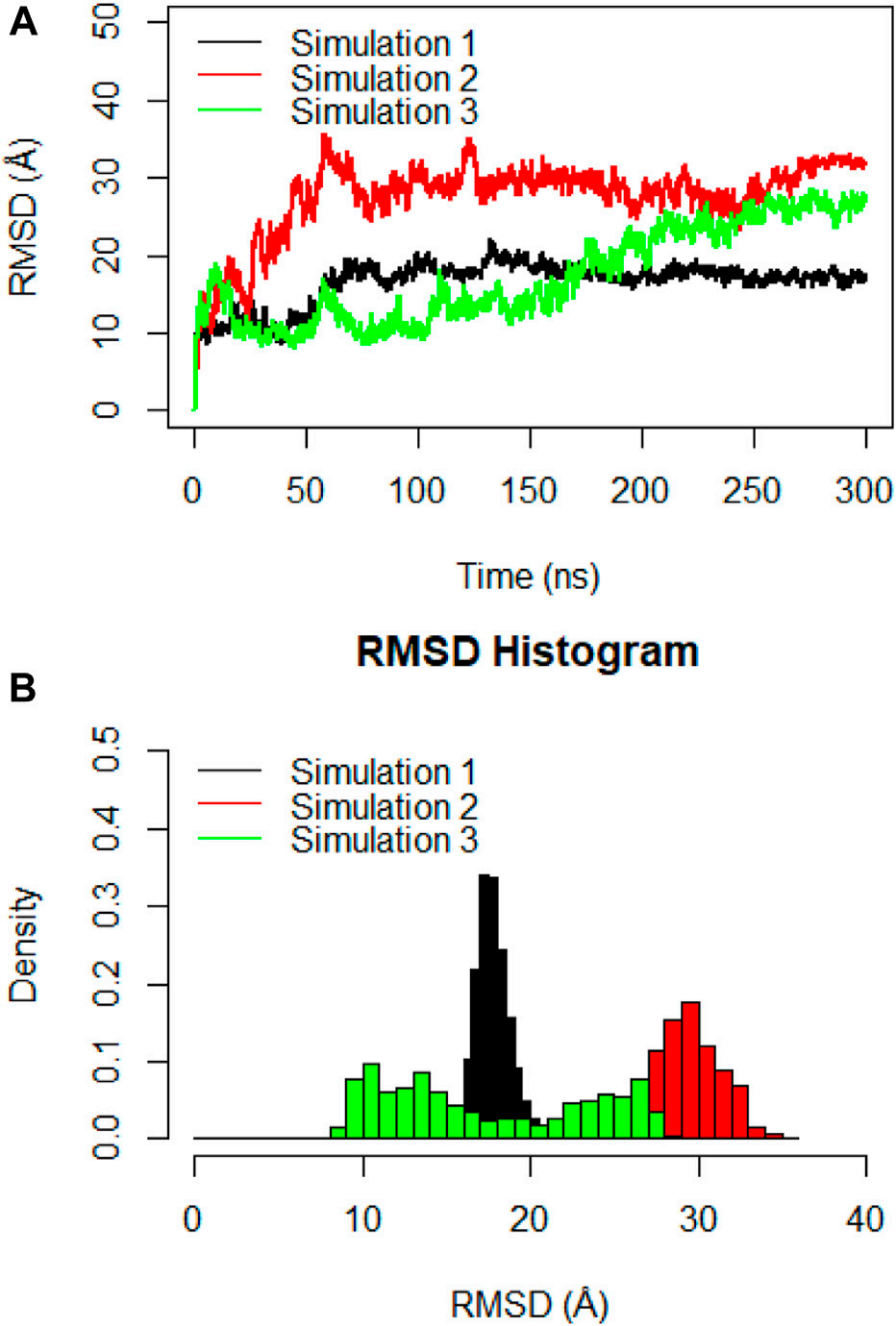
**(A)** Time-dependent RMSD plots of the monomeric spike-ferritin complex during three replicates of 300ns MD simulations. RMSD values were calculated for Cα atoms, with the X-axis indicating the Time (ns) and the Y-axis showing RMSD values in Å. **(B)** The histogram depicts the distribution of RMSD values for Cα atoms during a 300ns MD simulation. Values are based on calculations from all simulation replicates.

Molecular dynamics simulations were utilized to thoroughly examine the structural stability and flexibility of the monomeric spike-ferritin complex and its constituent parts. The RMSD analysis was employed to assess the global dynamics of the monomeric spike-ferritin structure. The general trend of time-dependent RMSD plots indicated an initial increase, followed by stabilization after 200 ns (Figure 5A). The average calculated RMSD values across three replicate simulations were 16.5 Å, 27.5 Å, and 17.4 Å, respectively. Notably, the observed high RMSD values are primarily attributed to the substantial size of the monomeric spike-ferritin nanoparticle structure (1,200 a. a) (Figure 2). These observations aligned with the expectation of reduced system stability due to the size of the spike monomer, which naturally results in a higher degree of conformational changes. The distribution patterns of calculated RMSD values derived from the RMSD histogram aligned with the findings of the time-dependent RMSD plots (Figure 5B). All replicates of the overall system revealed stable RMSD values with peaks at 16 Å. That is expected due to multiple domains within its structure (Figures 1, 2).

**FIGURE 6:**
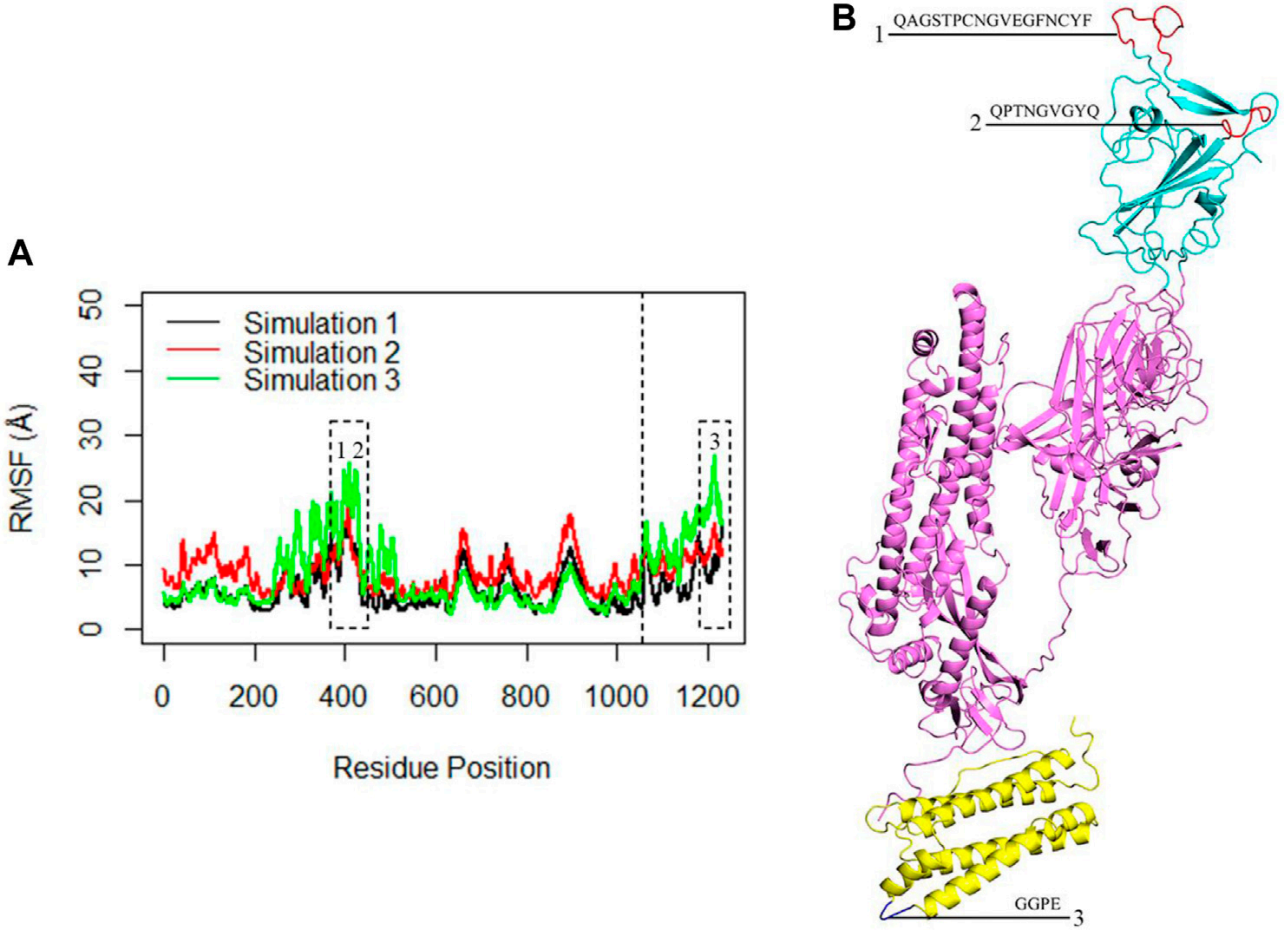
**(A)** The RMSF of the Cα atoms within the complex during three replicates of 300ns MD simulations. The X-axis corresponds to the residue number of the monomeric spike-ferritin, while the Y-axis shows RMSF values in Å. The dashed line separates the monomeric spike and ferritin amino acids. The first 1,058 amino acids correspond to the monomeric spike, while the subsequent 174 amino acids represent the ferritin. Dashed boxes highlight significant peaks in the RMSF plots. **(B)** The position of each loop associated with the peaks in the RMSF plots is presented.

The flexibility of the complex was further investigated through RMSF analysis, focusing on the Cα atoms per residue (Figure 6A). The average calculated RMSF values were 6.3 Å, 8.9 Å, and 8.4 Å for the three replicate simulations. These findings corroborated the insights gained from RMSD analysis and indicated that the monomeric spike-ferritin complex is highly stable. The RMSF plots revealed multiple peaks, which indicated the presence of distinct flexible loops and their dynamic motions within the structure. Specifically, the RBD region exhibited prominent peaks at residues (476–490) and (498–507) (Figure 6B), highlighting the dynamic motions within these specific loops. Similarly, fluctuations were observed in the ferritin structure, particularly at residues (155–158), suggesting the dynamic nature of the loops within its structure (Figure 6B). However, it is important to note that while these loops were flexible, they did not directly interfere in the spike-ferritin interface and thus the stability of the designed vaccine may not be affected. Loops one and 2, which were located in the interface of RBD with ACE2, were far from the monomeric spike interface with ferritin, and, therefore, would not affect the stability of the modeled vaccine. Loop three in ferritin was also located far from the interface. Thus, despite the flexibility indicated by the peaks in the RMSF plots, these loops did not influence the stability of the designed vaccine.

The Principal Component Analysis (PCA) and its results were useful in revealing the structural changes in the monomeric spike-ferritin complex (Supplementary Figure S8). PCA, which effectively reduces the dimensionality, was performed to identify the most significant fluctuation modes of the complex. Notably, the percentage of variance captured by PC1 and PC2 of the system amounted to 73.23% and 13.88%, respectively. These findings directly demonstrated that structural fluctuations are noticeable in the systems.

**FIGURE 7:**
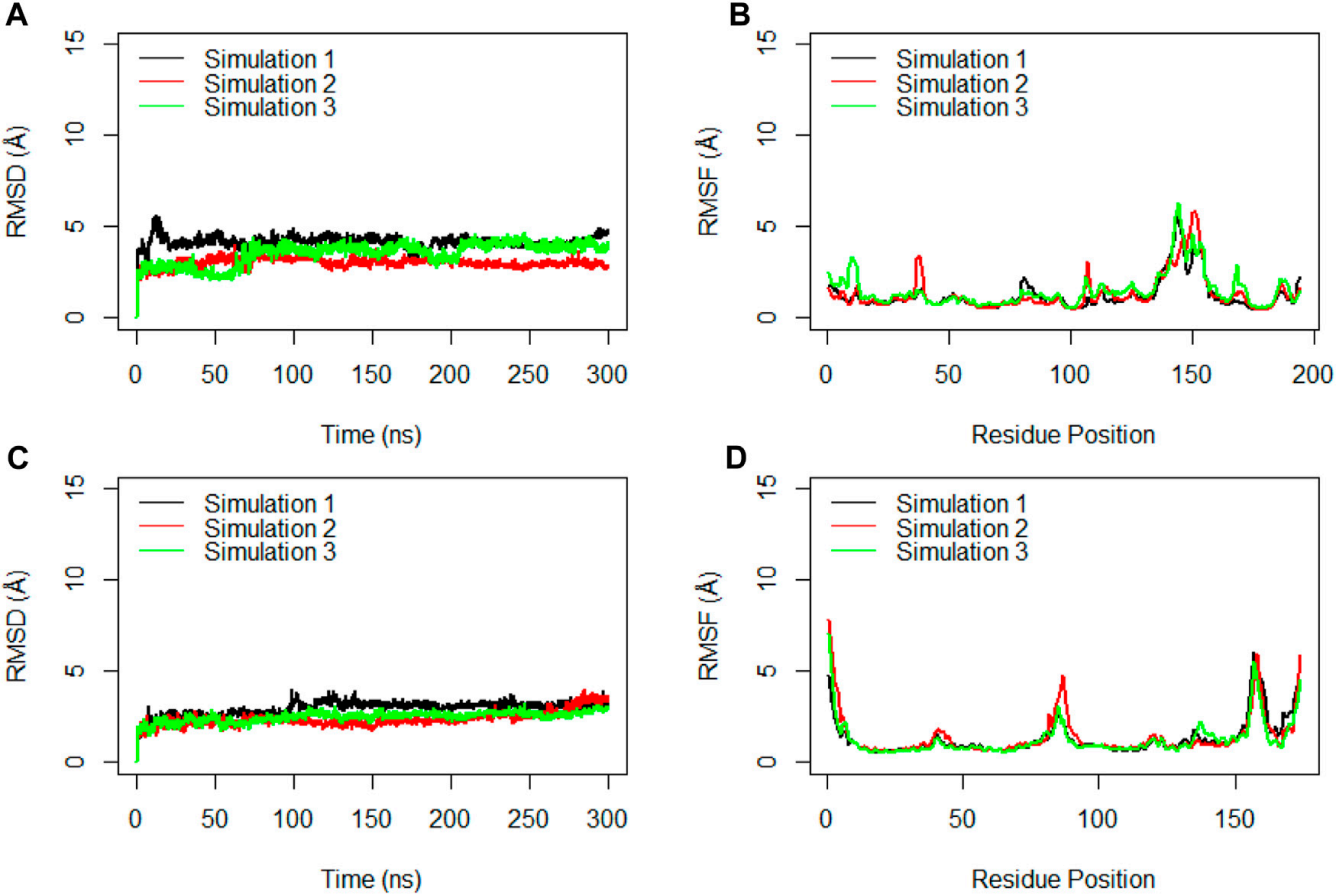
RMSD **(A)** and RMSF **(B)** for the Cα atoms of the Receptor Binding Domain (RBD) during the simulation time. RMSD **(C)** and RMSF **(D)** for the Cα atoms of the ferritin during three replicated of 300ns MD simulations. Values are based on calculations from all simulation replicates.

To gain deeper insights into the interactions within the RBD of the monomeric spike and ferritin, separate RMSD and RMSF analyses were conducted for these components (Figure 7). Notably, three replicate simulations displayed rather consistent structural stability for RBD, with average RMSD values of 4.2 Å, 3.0 Å, and 3.6 Å (Figure 7A). Suggesting that when RBD is present within the full ectodomain spike monomer, it remains structurally stable, as evidenced by the RMSF analysis as well. The average RMSF values obtained for RBD were 1.3 Å, 1.3 Å, and 1.5 Å, respectively (Figure 7B).

Similarly, the examination of ferritin across three replicates yielded RMSD values averaging at 3.0 Å, 2.4 Å, and 2.5 Å (Figure 7C). These RMSD plots indicated the significant stability of ferritin when it creates a complex with the full ectodomain spike monomer.

### Analysis of the secondary structure of the monomeric spike

**FIGURE 8:**
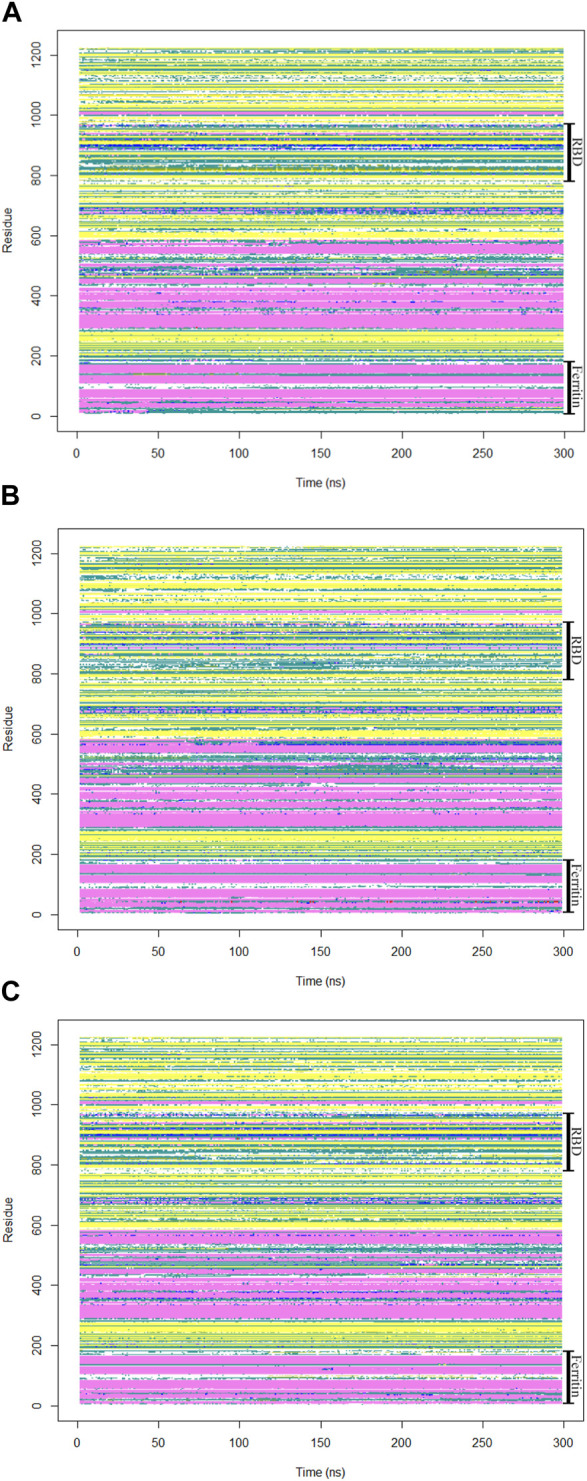
The secondary structures of all residues in the monomeric spike-ferritin complex were analyzed during a 300 ns duration of MD simulation for all replicates **(A–C)**.

To gain insights into crucial changes in protein structure during the molecular dynamics simulations, the secondary structures were calculated for the trajectories (Figure 8). The secondary structure analysis of the monomeric spike-ferritin complex did not show any significant changes in its dynamics, indicating overall stability of the complex.

In summary, it was demonstrated that the RBD and ferritin components within the monomeric spike-ferritin complex maintain strong stability. These findings have significant implications, suggesting that the full ectodomain spike monomer, despite its substantial size, holds promise for vaccine development against SARS-CoV-2.

**FIGURE 9:**
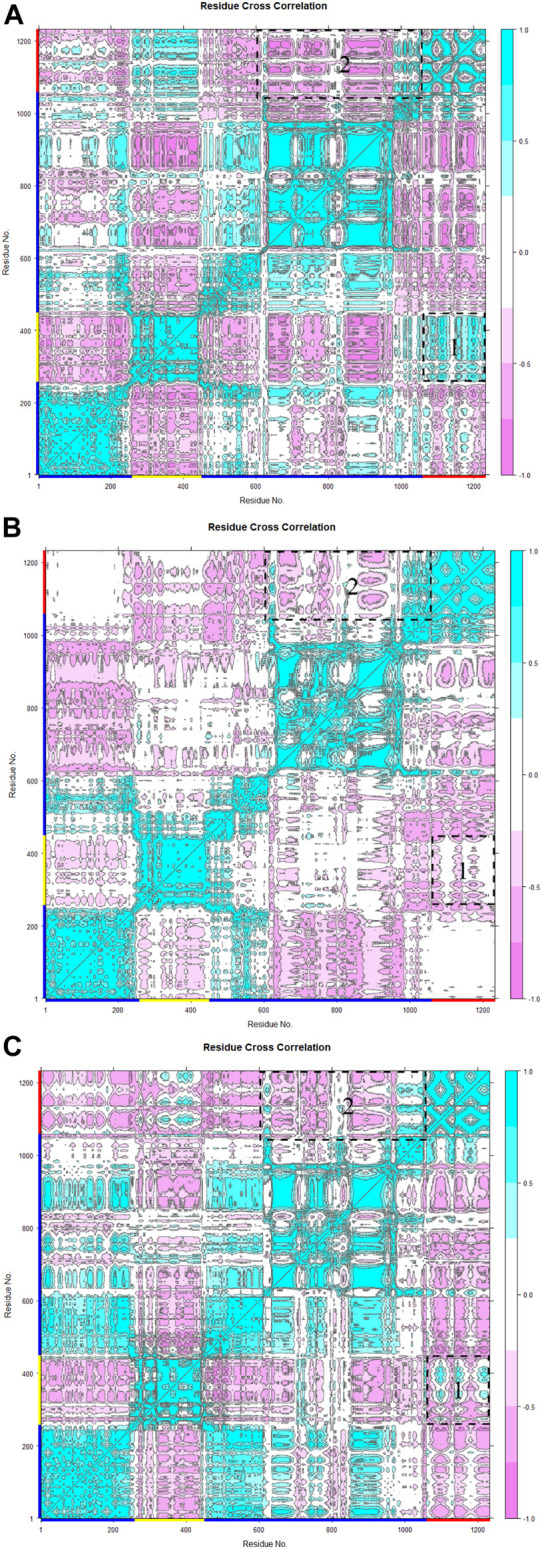
Residue cross-correlation matrix of monomeric spike-ferritin was calculated over three replicates **(A–C)** of 300 ns MD simulations for Cα atoms. The color bar on the right side displays correlation values, ranging from −1 (anti-correlated) to 1 (correlated). Pink represents anti-correlated motions and cyan indicates correlated motions. Residues of monomeric spike, RBD, and ferritin are shown with blue, yellow, and red lines respectively on the *x* and *y*-axes. Motions between RBD-ferritin (box 1) and S2-ferritin (box 2) are shown with dashed boxes.

**FIGURE 10:**
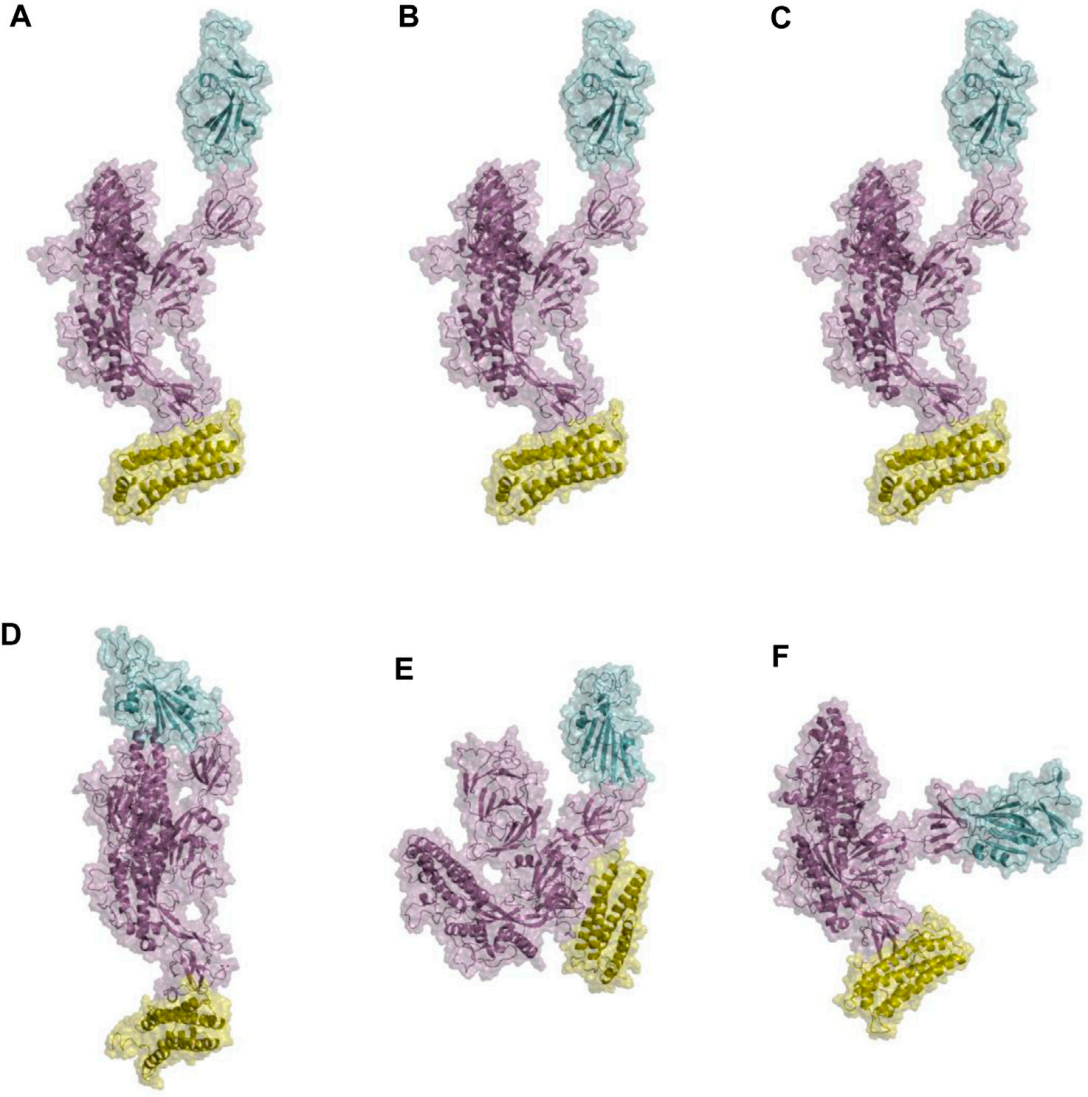
Structural presentations of monomeric spike attached to one unit of ferritin. Panels **(A–C)** represent the initial conformations of three replicate simulations. Panels **(D–F)** are final conformations. spike monomer, RBD, and ferritin are shown in violet, cyan, and yellow cartoon plus transparent surfaces respectively.

In the analysis of spike-ferritin interactions, the cross-correlation matrices of the Cα atoms were calculated in all simulations (Figure 9). Positive and negative values were used to denote correlated and anti-correlated motions, respectively. Anti-correlated motions occurring between spike and ferritin supported the stable interaction between the two proteins. It was also observed that when the spike becomes compact, the anti-correlation between RBD and ferritin grows as they come closer to each other (Figure 9B, C; Figure 10E, F).

Furthermore, visualizations of the dynamics indicated that the observed anti-correlated motions would not disturb the ‘‘up’’ active conformation of RBD (Figure 10). This is crucial because RBD needs to remain in an upward orientation to be accessible to ACE2.

**FIGURE 11:**
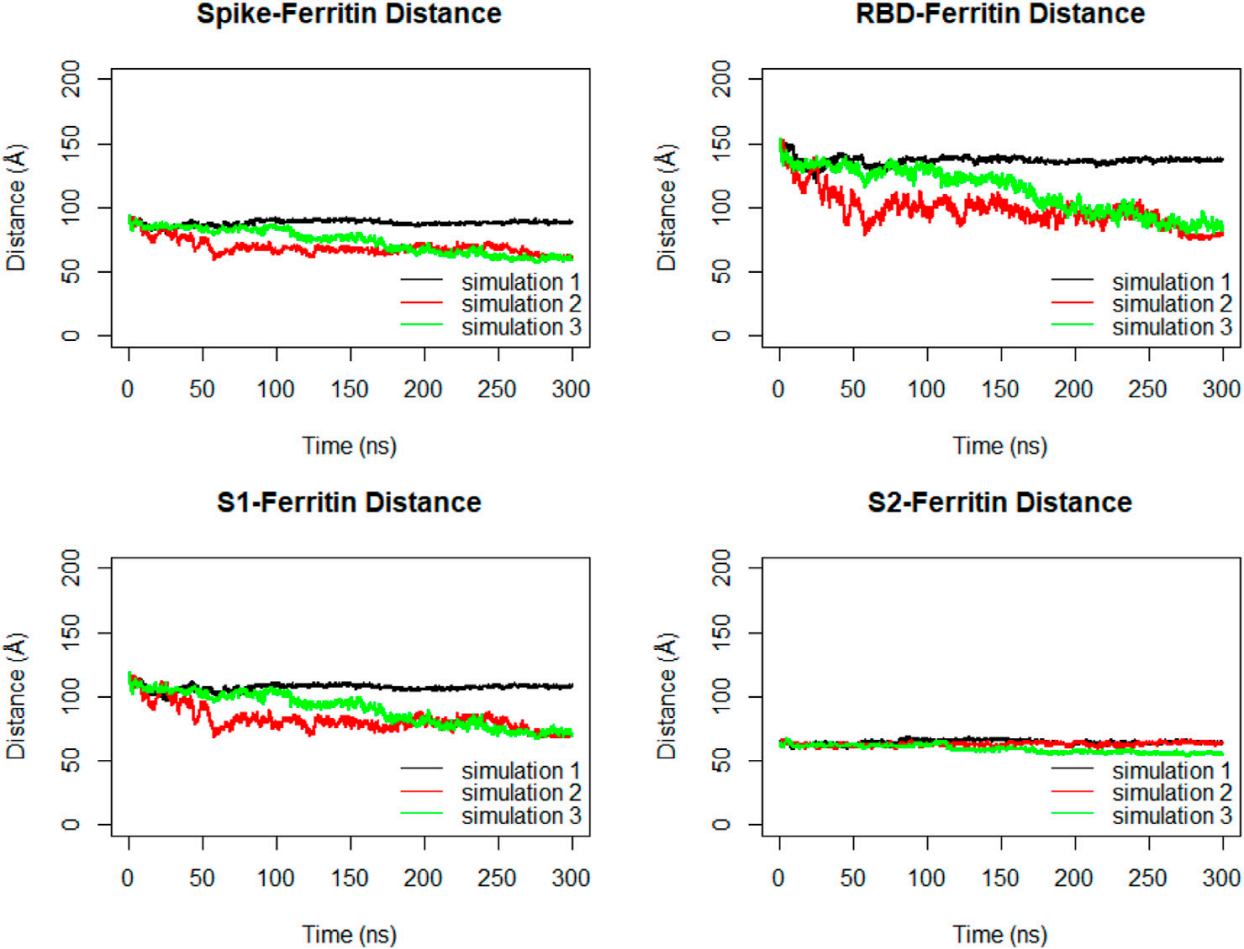
Distance between center of masses of ferritin-spike, ferritin-RBD, ferritin-S1, and ferritin-S2 within three replicated of 300 ns MD simulations. Values are based on calculations from all simulation replicates.

The distance between the center of masses of ferritin and specific regions of the spike protein, including RBD, S1, and S2, was calculated over three replicates of 300ns MD simulations (Figure 11). In general, a descending trend was evident in the calculated distances during the simulation. Among calculated distances, S2-ferritin exhibited the least variation, while RBD-ferritin showed the greatest change in the overall trend. The observed decline in the distance was supported by the visualization of dynamics, indicating that the spike becomes more compact, with the S1 and S2 subunits of the spike approaching each other. However, the RBD maintains its upward conformation consistently throughout the entire simulation time (Figure 10).

**FIGURE 12:**
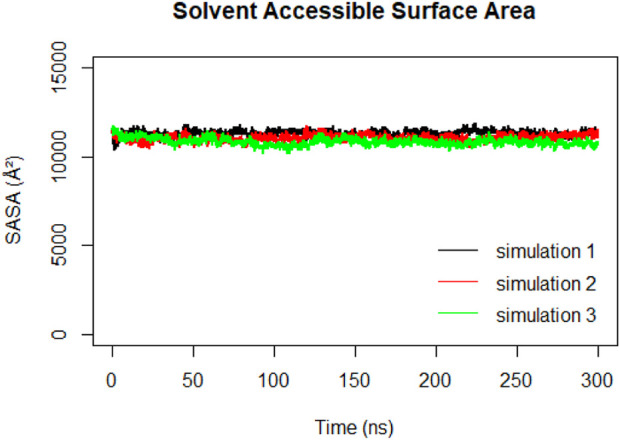
Solvent Accessible Surface Area (SASA) of the RBD during 300ns MD simulations.

Solvent Accessible Surface Area (SASA) calculations were performed on the RBD to track changes in its exposure and accessibility over time (Figure 12). The stable trend observed in the RBD SASA values suggested that despite structural changes in the spike protein, the RBD maintains its accessibility to the ACE2 receptor. This indicates that the RBD’s binding ability to the ACE2 receptor remains relatively constant, even during the compaction that happens to the spike protein.

### Immune simulation of monomeric nanoparticle vaccine model

**FIGURE 13:**
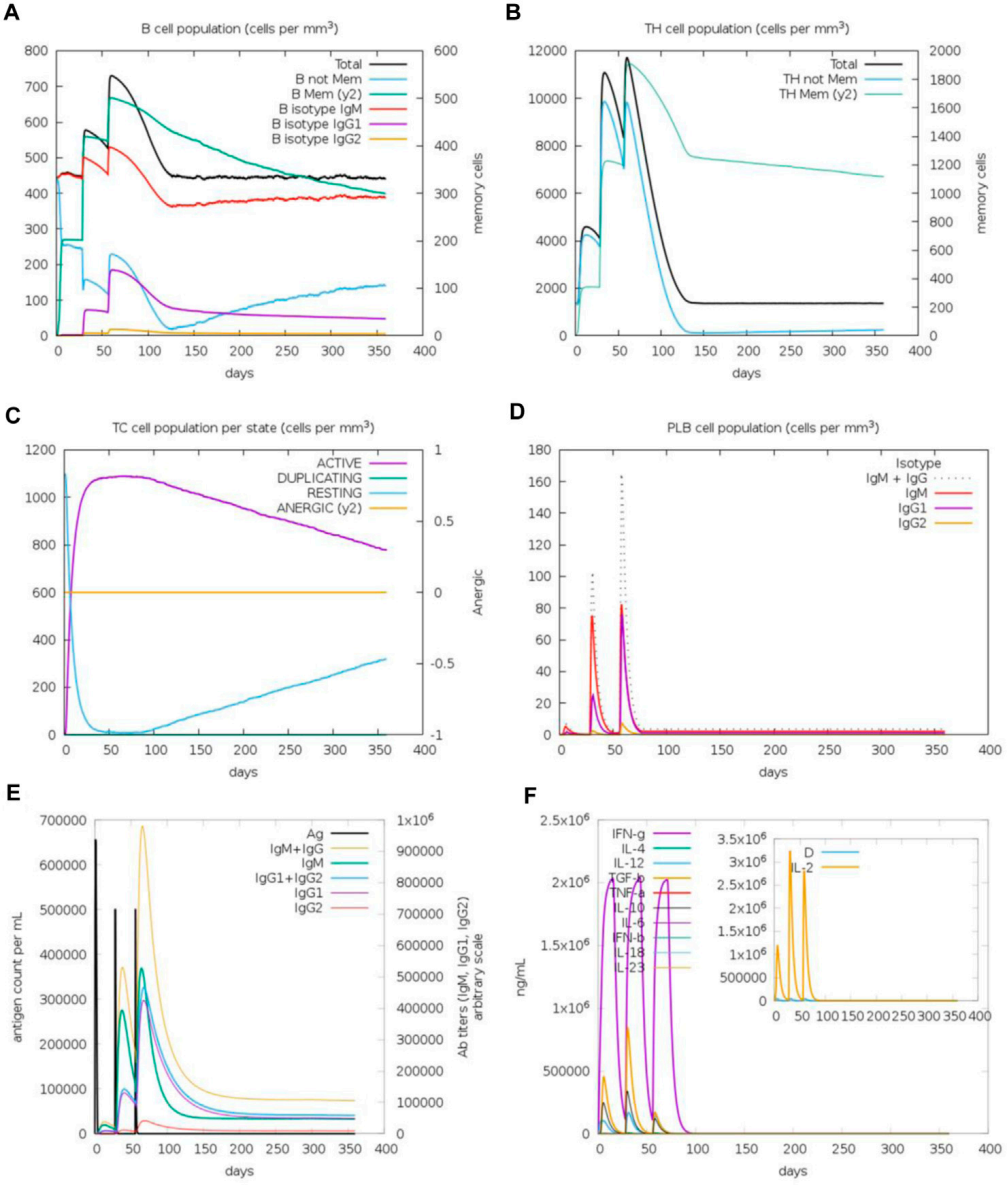
Immune response simulations of monomeric spike-ferritin nanoparticle vaccine with 1 month intervals. **(A)** B cell population, **(B)** CD4^+^ T cell population, **(C)** CD8^+^ T cell population, **(D)** Plasma B cell population, **(E)** Humoral immunity response, **(F)** Concentration of cytokines and interleukins.

The immunogenic profile of the monomeric spike nanoparticle vaccine candidate was obtained from the C-IMMSIM server. The vaccine was injected in three doses over 2 months with 30-day intervals (Figure 13). It was shown that the modeled vaccine was able to elicit both humoral and cellular mediated immune responses. Following each vaccine dose, there was an increase in B cell population, indicating that the vaccine effectively stimulated the immune system and led to a more robust antibody response (Figure 13A). Additionally, the CD4^+^ T cell population peaked after the third injection (Figure 13B). Investigation of the CD8^+^ T cell population revealed a higher population of active cells compared to anergic cells (Figure 13C). Furthermore, the plasma cell population revealed a substantial increase (>80 cells per mm) following the third dose, suggesting robust antibody production (Figure 13D). The antibody response to the monomeric nanoparticle vaccine model (Figure 13E) exhibited a slight increase in the graphs after the first two doses. From the second dose onwards, there was a rise in antibody secretions attributed to memory T lymphocytes. An upward trend of IgM and IgG antibody titer was observed after the third injection, alongside a decline in antigen levels. Additionally, elevated levels of cytokines such as IFN-γ and IL-2 were observed (Figure 13F). Clearance of antigenic molecules was demonstrated after three doses of vaccination. There was also a significant increase in the populations of both B and T memory cells, which remained elevated for several months.

Upon analysis of two additional simulations conducted with different random seeds (Supplementary Figures S5, S6), it became evident that the immune responses observed across all three simulations were comparable. Minor variations were noted, with indications of improved immune responses observed in some cases. Importantly, the pattern of immune system stimulation remained consistent across all three simulations.

### HLA variants and immune response to monomeric nanoparticle vaccine

The adaptive immune response to SARS-CoV-2 relies on the activity of T cells, wherein MHC molecules play a crucial role in processing and presenting antigenic peptides to T cells, initiating immune responses (Lin et al., 2023). Human MHC molecules, also referred to as Human Leukocyte Antigen (HLA), are divided into two classes. MHC class I (HLA-A, HLA-B, and HLA-C) present peptides from endogenous sources to CD8^+^ T cells, while MHC class II molecules (HLA-DR, HLA-DP, and HLA-DQ) predominantly process exogenous peptides, presenting them to CD4^+^ T cells (Traherne, 2008). The diversity in HLA alleles contributes to variations in peptide processing and presentation, thereby influencing immune responses (Wolday et al., 2023).

In order to ensure the effectiveness of the designed vaccine against different variants of HLA, the prediction of T cell epitopes on the monomeric spike protein of the vaccine was performed. However, due to the high diversity in HLA alleles, assessing all variants was not feasible. Instead, the study focused on investigating the two most polymorphic MHC genes. The HLA-B gene, with 1,077 reported alleles, is recognized as the most polymorphic among MHC class I genes, while the HLA-DRB1 gene, with 669 alleles, stands out as the most polymorphic among MHC class II genes (Shiina et al., 2009; Aliseychik et al., 2018). The analysis of monomeric spike peptides processed by HLA-B variants revealed notable findings. For each of the examined variants, 3,620 peptides were predicted. These peptides were then assessed based on their affinity for binding to HLA-B molecules. The prob score distribution of monomeric spike peptides processed by five common HLA-B variants revealed distinct patterns (Supplementary Figure S9). Overall, variant HLA-B*57:01 exhibited the highest prob score distribution, with three peptides scoring as high as 1, indicating a strong probability of binding to HLA-B molecules. Following closely were variants HLA-B*53:01 and HLA-B*44:03, which also showed relatively high prob score distributions. In contrast, the low prob score distribution and median for variants HLA-B*35:01 and HLA-B*07:02 suggested a lower probability of binding processed peptides to HLA-B molecules. Remarkably, despite the diversity in HLA-B alleles, peptides with a high probability of binding to HLA-B molecules (>70%) were identified for all of five most common HLA-B variants. Further exploration involved predicting peptides that bind to MHC class II variants. A thorough examination assessed the binding affinity of predicted peptides across 13 alleles of HLA-DRB1 and its paralogues: HLA-DRB3, HLA-DRB4, and HLA-DRB5 (Supplementary Figure S10). Remarkably, a consistent pattern was observed across all alleles. While the majority of predicted peptides exhibited a binding affinity distribution ranging from 0 to 0.1, it is noteworthy that all alleles (except for HLA-DRB4*01:01) presented peptides with a high probability (>60%) of binding to MHC class II molecules. These observations underscore the robustness of the immune system’s response to the monomeric nanoparticle vaccine across both MHC class I and class II pathways, despite variations in MHC alleles.

## Discussion

This study highlighted the role of *in silico* structural modeling in rapidly generating information on novel viruses to help predict their behavior and aid in countermeasure development. The main focus of this work was on the SARS-CoV-2 which caused the extensive pandemic of COVID-19. The structural spike protein of the virus has been revealed as the major component responsible for its pathogenicity (Du et al., 2009; Yang et al., 2020). This protein plays a key role in facilitating the virus’s entry into human cells by binding to a receptor called ACE2 (Hoffmann et al., 2020; Walls et al., 2020). Upon attachment to ACE2, the structural spike protein enables the virus to infect the cell, leading to the development of the disease. The RBD is the most immunogenic domain interacting directly with ACE2 (Fehr and Perlman, 2015; Shereen et al., 2020); thus, the focus of vaccine development in this study was on presenting this particular domain within a monomeric spike protein structure. Ferritin, primarily a cytosolic protein serving as a nanocage for iron storage, is also secreted in small amounts in the serum as an iron carrier (Andrews et al., 1992). In this study, nanoparticle ferritin was employed to display monomeric spikes as an immune system stimulant. The ferritin assembly used in this study lacked iron ions, addressing concerns about ferritin releasing iron into the bloodstream or tissues and causing potential interference. Ferritin nanoparticle vaccines, previously created to combat other infections, are now designed to fight SARS-CoV-2 and produce significant immunogenicity (Sliepen et al., 2015; Wang et al., 2017; Kalathiya et al., 2021; Powell et al., 2021; Wang et al., 2021; Wuertz et al., 2021). In this study, structural molecular modeling was employed to construct and explore the dynamics of the ferritin nanocage monomeric spike SARS-CoV-2 vaccine at the atomistic level for the first time. The vaccine was developed using the monomeric spike as a crucial viral component in its construction. Through molecular docking and consequently placing the ferritin units in an icosahedral full-cage structure, a monomeric spike model of the ferritin nanoparticle vaccine was obtained. Additionally, molecular dynamics simulations were conducted, providing a comprehensive analysis of the dynamic behavior and interactions within the monomeric spike-ferritin complex.

According to the analysis of trajectories, the monomeric spike-ferritin complex revealed overall stability and structural integrity, which are crucial for its functionality. Additionally, the native conformation of the antigen, necessary for immune recognition, remained preserved. Furthermore, the proper orientation of spike protein monomers within the ferritin unit allowed critical epitopes to be exposed to the immune system. The components of the vaccine demonstrated proper folding, maintaining their functional structures. Simulation of the immunity indicated that the monomeric spike model would stimulate cellular and humoral immunity in the human body and reduce the antigen amount. In addition, 2,458 peptides were identified with a high probability (>70%) of binding to MHC molecules of the five most common HLA-B variants, indicating their potential as epitopes for immune response stimulation. Notably, the HLA-B*57:01 variant demonstrated significantly higher peptide binding affinity compared to other HLA-B variants, presenting an opportunity for personalized vaccine design. Specifically, populations with a high frequency of the HLA-B*57:01 allele could benefit from a customized vaccine formulation designed to maximize immune response efficacy. Also, all examined HLA-DRB1 alleles exhibited peptides with a high probability (>60%) of binding to MHC class II molecules, demonstrating the potential for robust immune activation. Finally, the reliability of the modeled interfaces was also investigated by examining the mutation sites related to variants of concern, including Alpha, Beta, Gamma, and Omicron, on the spike protein. The results revealed that none of these mutations occurred at the interfaces of the model. Also, in a study that predicted possible mutation locations, three out of five mutations occurred in the previous variants, and two more mutations, including Y489 and T500, are expected to occur in the following variants. However, these threatening mutations are not only not located at the binding surface of ferritin and spike but also far from it (Mohammad et al., 2021; Alkhatib et al., 2022).

In previous studies, the efficacy of the RBD-ferritin nanoparticle vaccine’s immunogenicity has been established (Kalathiya et al., 2021; Masoomi Nomandan et al., 2022). The studies successfully developed RBD-based subunit vaccines employing ferritin protein nanocages. Their findings highlight the vaccine’s efficacy in presenting RBD, ensuring stability, and triggering a robust immune response. These efforts to develop a SARS-CoV-2 nanoparticle vaccine have predominantly focused on utilizing the RBD. However, experimental evidence has indicated that the full-length spike may be more effective (Powell et al., 2021). This current study aimed to investigate the suitability of using spike monomer rather than just RBD in the design of subunit vaccines. Utilizing the full-length ectodomain monomeric spike protein ensures that various epitopes outside the RBD region are also presented to the immune system, potentially offering protection against different variants of the virus. In this study, it was observed that the monomeric spike–ferritin complex, which is a large system with about 1,200 a. a, is highly stable compared to the RBD-ferritin vaccine construct. Immunogenicity results were comparable to those of the RBD-ferritin vaccine (Masoomi Nomandan et al., 2022), indicating that the designed vaccine is capable of producing an immune response. In general, the results obtained from this vaccine supported the potential effectiveness of utilizing the monomeric spike in nanoparticle vaccine designs.

As mentioned in several previous studies (Kalathiya et al., 2021; Powell et al., 2021), multivalent presentation of SARS-CoV-2 spike on ferritin can notably enhance the elicitation of neutralizing antibodies, constituting a viable strategy for single-dose vaccination against COVID-19. Although ferritin nanoparticle vaccines have already demonstrated promising clinical benefits against several microbial infections, including SARS-CoV-2, the molecular mechanism of these nanoparticle assemblies and the underlying atomistic interactions were poorly understood.

The SARS-CoV-2 spike protein is heavily glycosylated (Shajahan et al., 2020; Yasunori et al., 2020; Gong et al., 2021). Thus, the glycosylation of the monomeric spike in the SARS-CoV-2 nanoparticle vaccine holds significant potential to influence the immune response elicited by the designed vaccine in various ways. Glycosylation has been identified as an important factor impacting protein folding, stability, and ligand binding (Lee et al., 2015; Watanabe et al., 2019; Gong et al., 2021; Rahnama et al., 2021). Proper glycosylation of the vaccine spike protein is important for initiating a robust adaptive immune response, as it affects the folding of vaccine epitopes and antigen processing by immune cells (Reis et al., 2021). On the other hand, glycans have the potential to act as camouflage, concealing immunogenic epitopes from detection by the innate immune system (Casalino et al., 2020). This mechanism could potentially reduce the vaccine’s ability to induce effective immune responses against the virus. The simulations conducted in this research focused on structures without attached glycans. However, investigation of the N- and O-glycosylation profile of the spike protein ensured that there are no potential glycosylation sites within the ferritin-spike interface (residues: 1,147–1,136). Nevertheless, future studies could examine the presence and effects of glycans on the designed vaccine for a comprehensive understanding of vaccine immunogenicity. While *in silico* vaccine design offers invaluable insights, its efficacy and reliability depend on subsequent experimental confirmation through *in vitro* and *in vivo* studies. The study acknowledges the importance of integrating computational models with experimental data to advance the development of reliable and effective vaccines.

## Conclusion

Ferritin nanoparticle vaccines are one of the most effective vaccines that provide optimal immunity against the virus without threatening the patient’s health. In this work, molecular docking, molecular dynamics simulation, and immune response simulations were used to construct and evaluate the self-assembled ferritin nanoparticle and monomeric spike vaccine model for the first time. The monomeric spike-ferritin complex displayed significant stability, particularly in the RBD and ferritin. This stability was further supported with minimal changes observed in the secondary structure of spike and ferritin. Despite the observed compaction of the spike protein throughout the simulations, the accessibility of the RBD remained unchanged. Strong interactions between the monomeric spike and ferritin were indicated by their observed motions toward each other. Furthermore, the sustained upward conformation of the RBD potentially facilitated interaction with ACE2. The study on mutation sites related to variants of concern found no mutations at the modeled interfaces. Additionally, while potential future mutations (Y489 and T500) are predicted in subsequent variants, they are not positioned near crucial binding sites. These findings could be crucial in improving current ferritin nanoparticle vaccines as well as future nanoparticle vaccine formulations in both *in silico* and *in vitro* investigations.

## Data Availability

The raw data supporting the conclusion of this article will be made available by the authors, without undue reservation.
