# Supplementary material for: Computational design and investigation of the monomeric spike SARS-CoV-2-ferritin nanocage vaccine stability and interactions
Source: Front Mol Biosci. 2024 Jun 12;11:1403635. doi: 10.3389/fmolb.2024.1403635 (PMC11199398; doi:10.3389/fmolb.2024.1403635)
Supplement: Supplementary file 1 [file DataSheet1.docx]

**Supplementary Information**

**Computational Design and Investigation of the Monomeric Spike SARS-CoV-2-Ferritin Nanocage Vaccine Stability and Interactions**

Farnaz Garmeh Motlagh 1, Maryam Azimzadeh Irani 1*, Seyedeh Zeinab Masoomi Nomandan 1, Mohammad Assadizadeh 1

1 Faculty of Life Sciences and Biotechnology, Shahid Beheshti University, Tehran, Iran.

*Corresponding author email: m_azimzadeh@sbu.ac.ir

**Supplementary Tables**

**Table S1.** Molecular docking data of monomeric spike with ferritin, represented with ClusPro server outputs.

| **Cluster** | **Members** | **Weighted Score** |
| --- | --- | --- |
| 0 | 119 | -1618.4 |
| 1 | 99 | -1683.3 |
| 2 | 89 | -1608.6 |
| 3 | 66 | -1437 |

**Table S2.** Interactions between residues of the monomeric spike and ferritin are listed below.


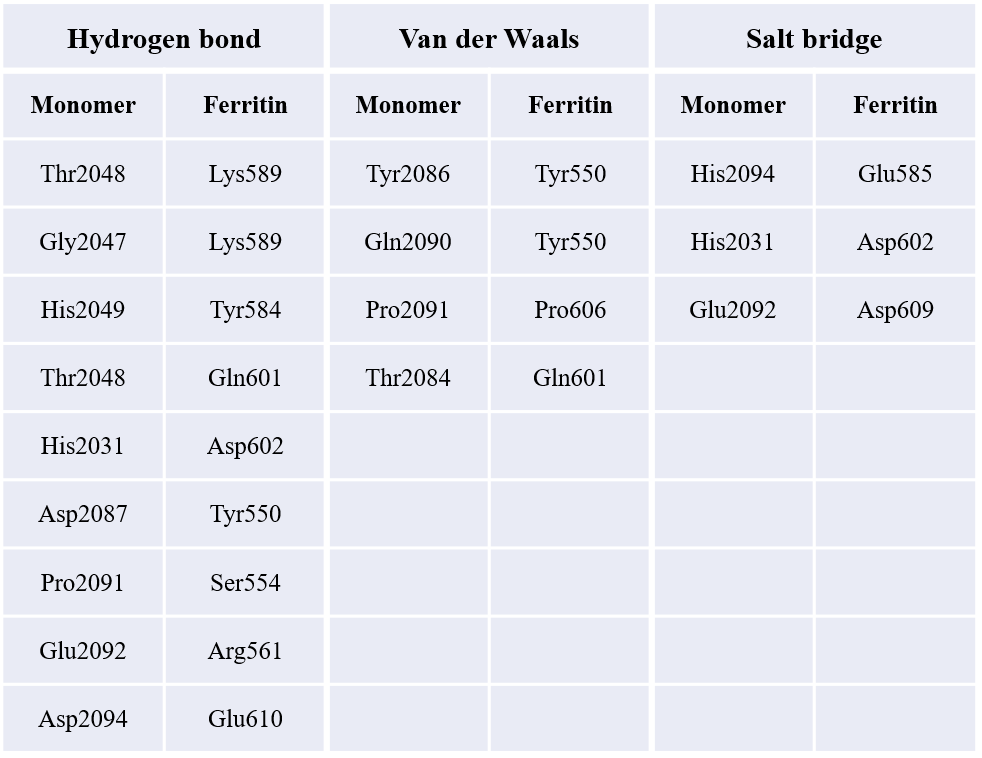


**Supplementary Figures**


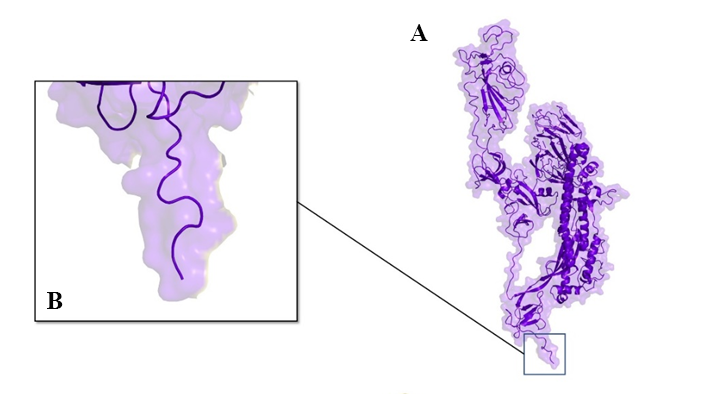


**Fig. S1.** (**A**) The monomeric spike in purple is shown in cartoon and surface representations. (**B**) The ferritin binding interface.


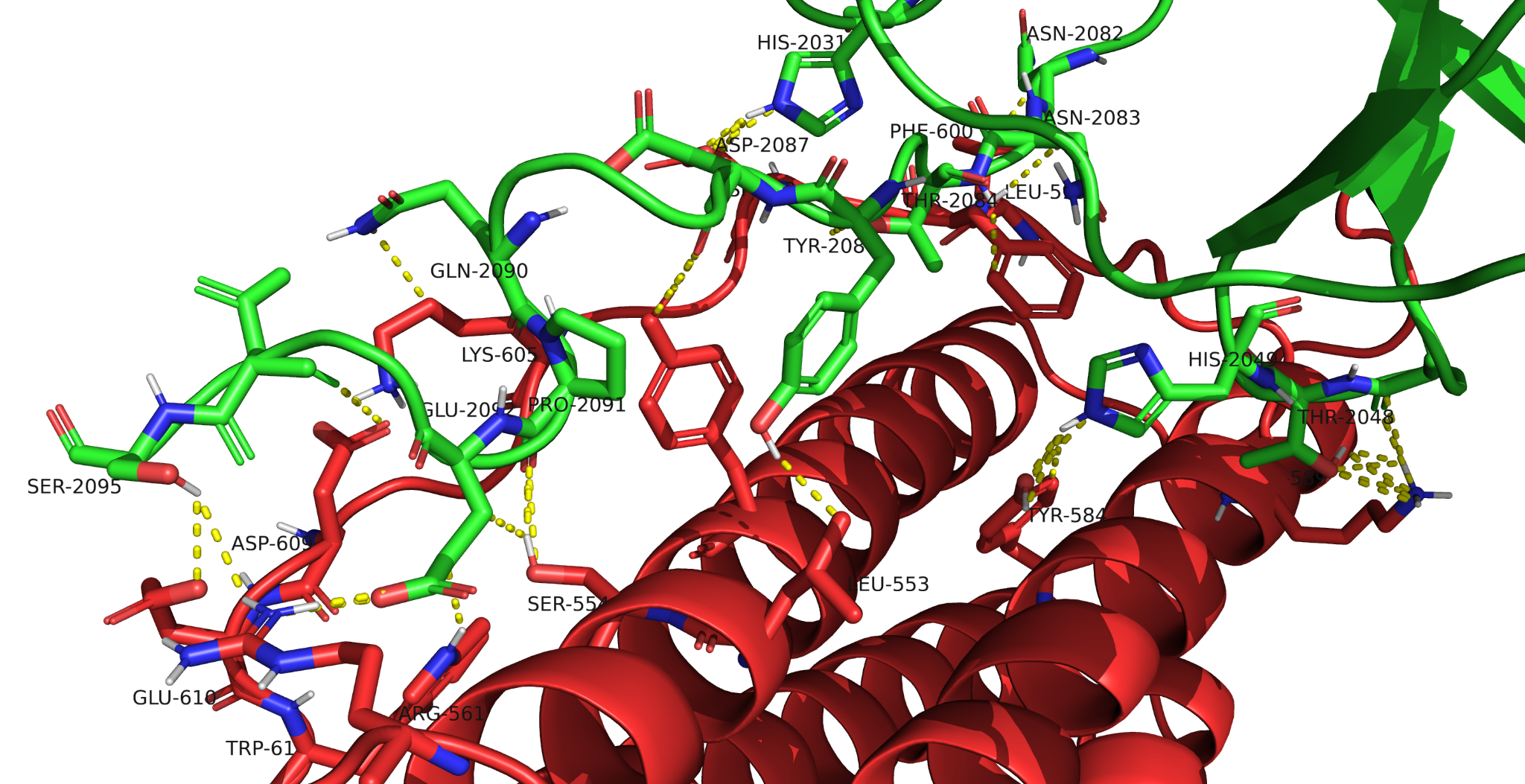


**Fig. S2.** Spike monomer bonds formed within a 3 Å with ferritin structure are shown with yellow dashed lines. Ferritin and monomeric spike are shown in red and green cartoons, respectively. The sticks show the amino acids that play a significant role in these bonds.


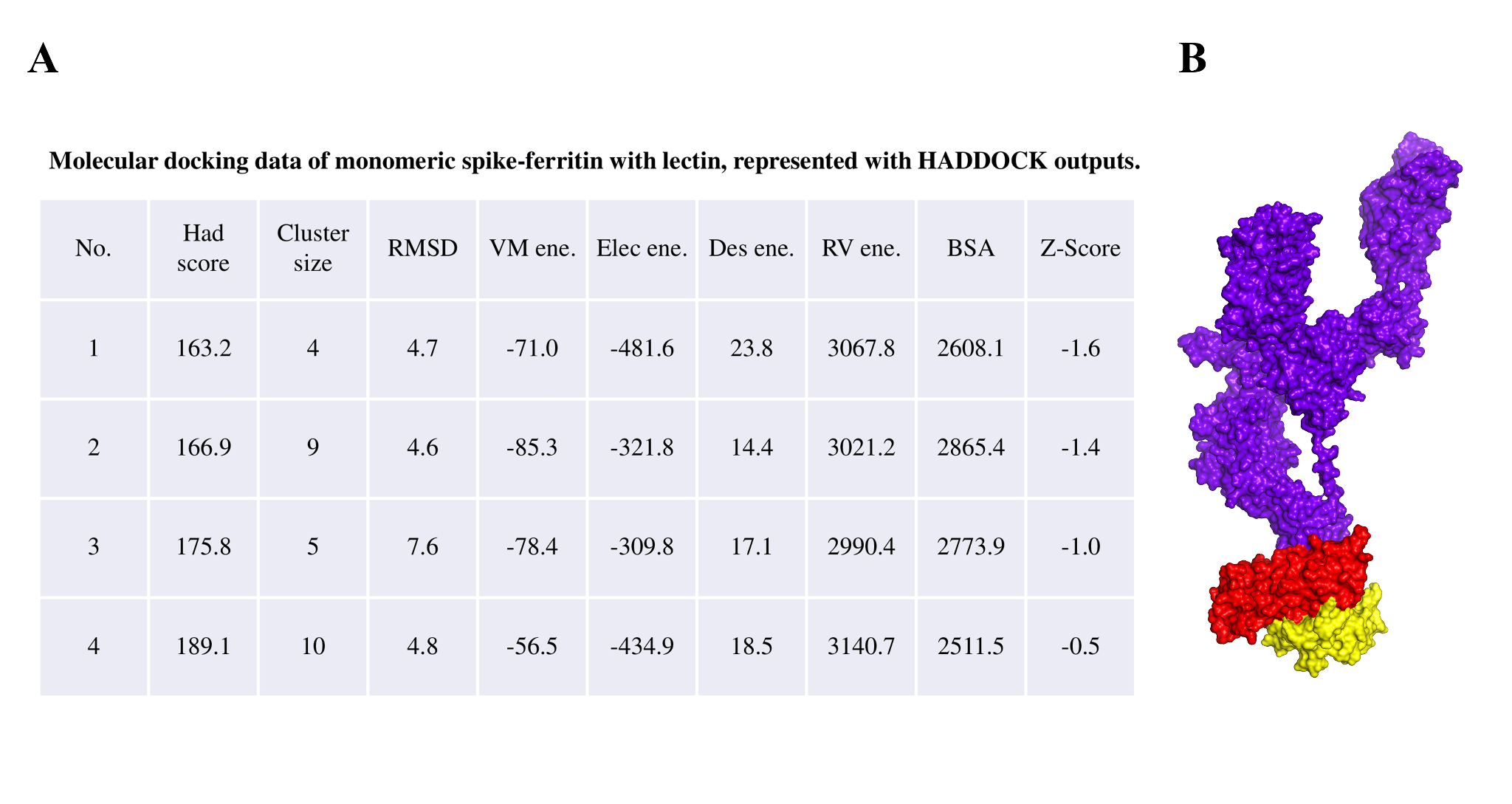


**Fig. S3.** (**A**) Molecular docking result of the monomeric spike-ferritin complex with lectin from the HADDOCK server. (**B**) The best binding pose is shown in the right panel, with the monomeric spike, ferritin unit, and lectin represented by purple, red, and yellow surfaces, respectively.

**
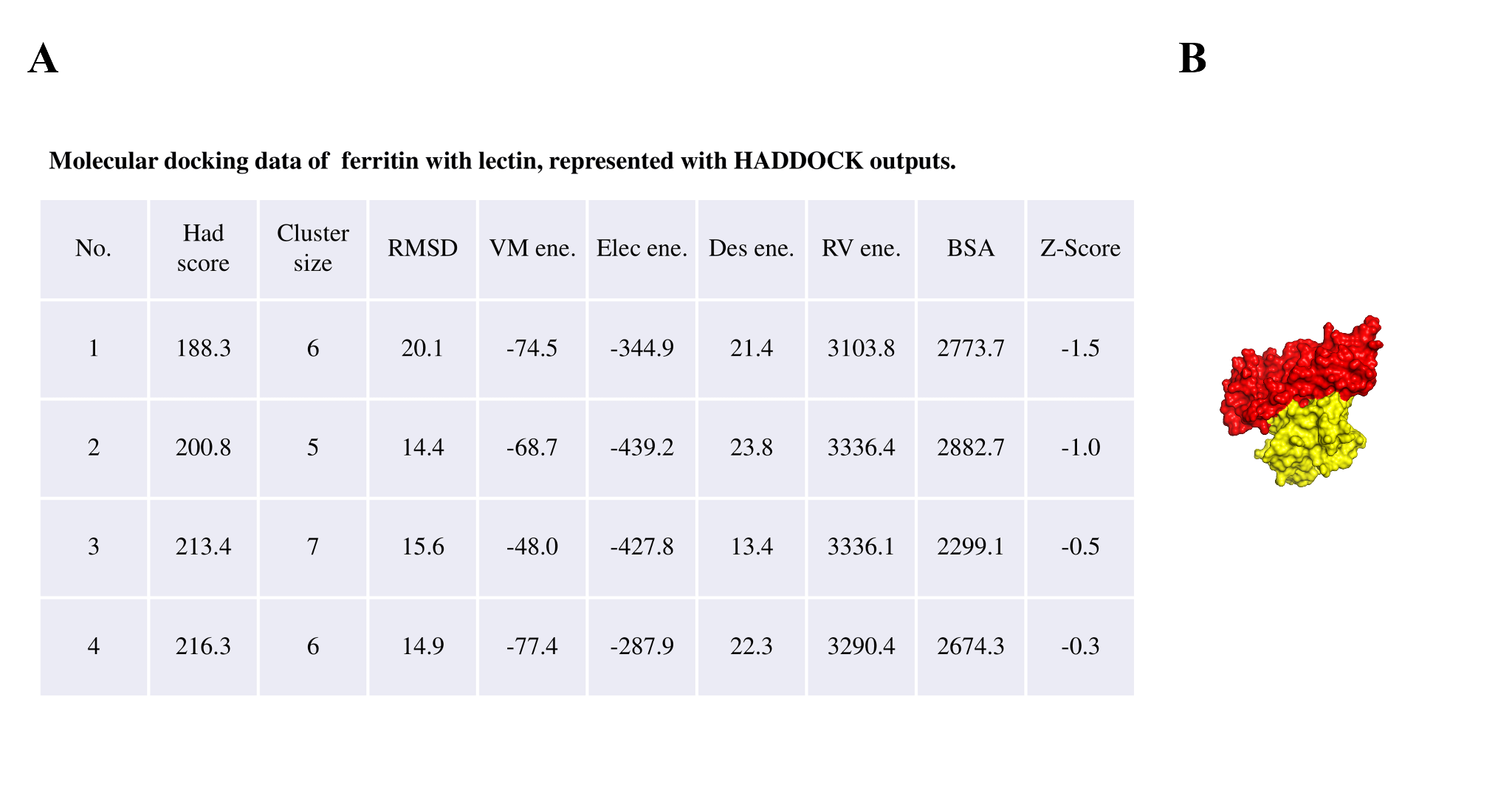
**

**Fig. S4.** (**A**) Molecular docking result of the ferritin unit with lectin from the HADDOCK server. (**B**) The best binding pose is shown in the right panel, with the ferritin unit and lectin represented by red and yellow surfaces, respectively.


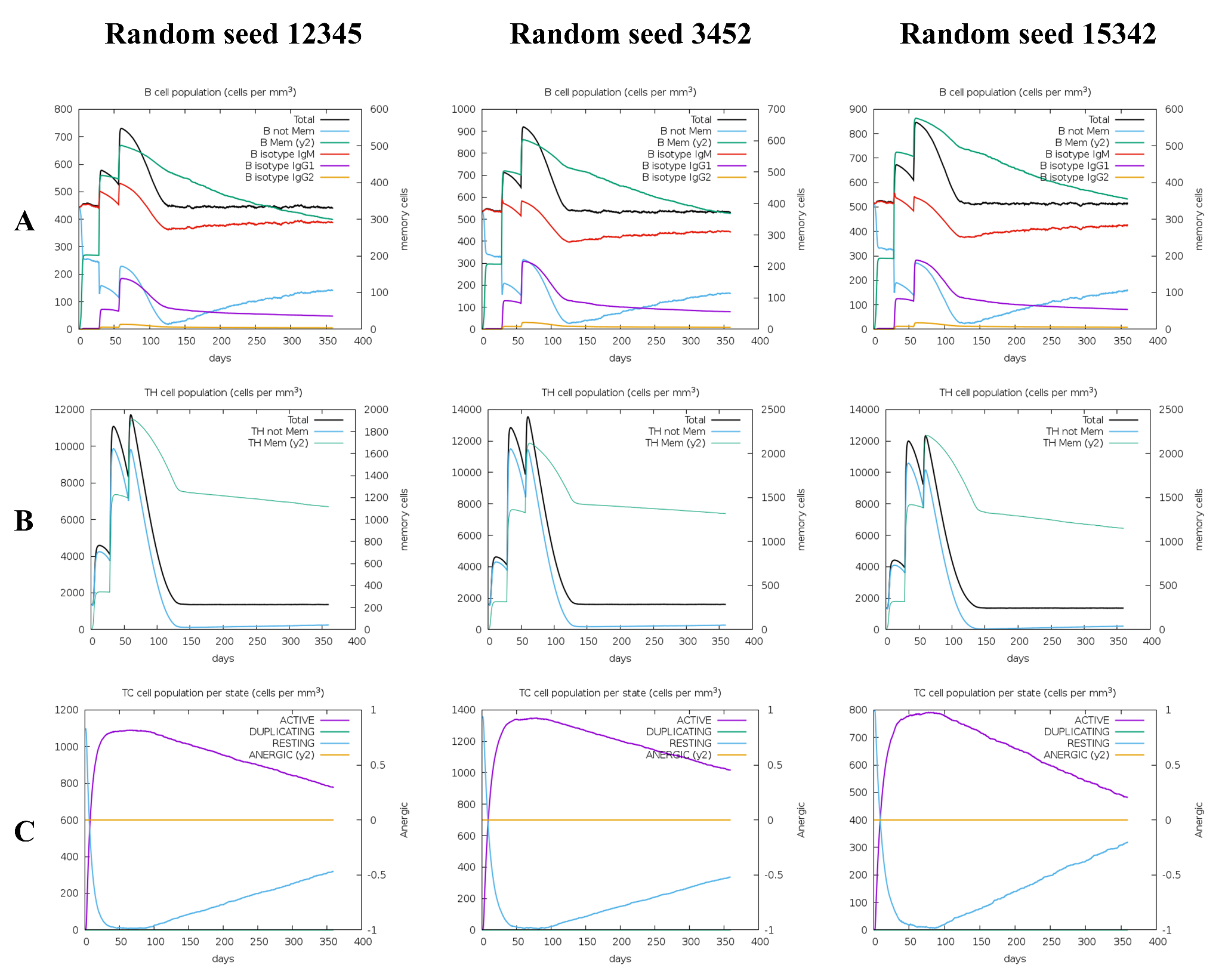


**Fig. S5.** Immune response simulations of the monomeric spike-ferritin nanoparticle vaccine were conducted with different random seed numbers. The initial seed number was set to 12345, while subsequent simulations used seed numbers 3452 and 15342. Panels (**A-C**) depict the B cell population, CD4^+^ T cell population, and CD8^+^ T cell population, respectively, obtained from these simulations.

**
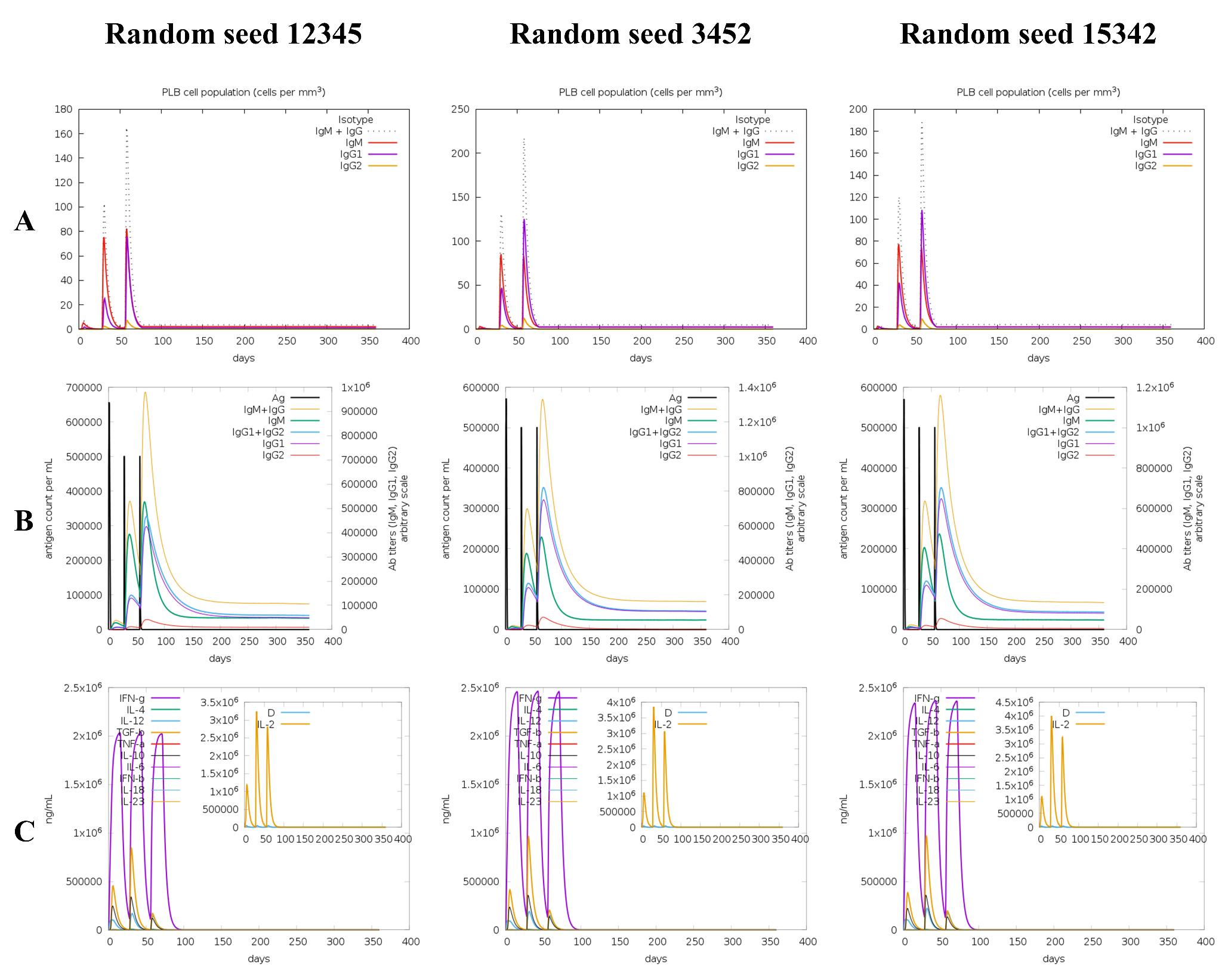
**

**Fig. S6.** Immune response simulations of the monomeric spike-ferritin nanoparticle vaccine were conducted with different random seed numbers. The initial seed number was set to 12345, while subsequent simulations used seed numbers 3452 and 15342. Panels (**A-C**) depict the plasma B cell population, humoral immunity response, and concentration of cytokines and interleukins, respectively, obtained from these simulations.


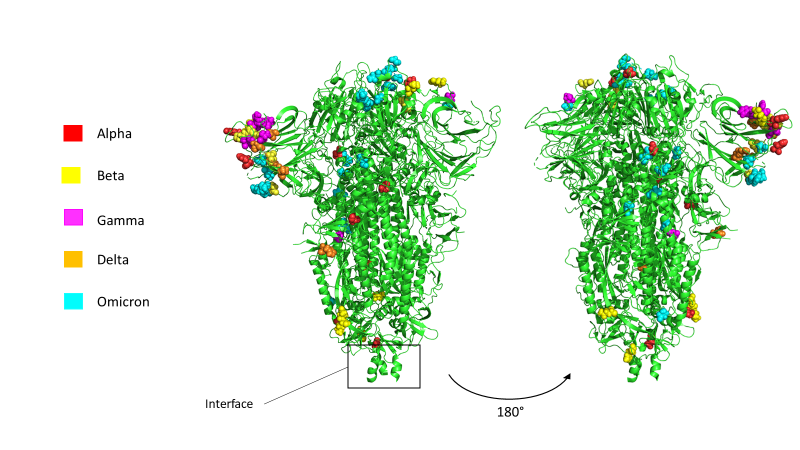


**Fig. S7.** Spike mutations that occurred in variants of concern are shown in colored spheres. Red for the Alpha variant, yellow for the Beta variant, magenta for the Gamma variant, orange for the Delta variant, and cyan for the Omicron variant. The binding interface of the spike is shown in the box.


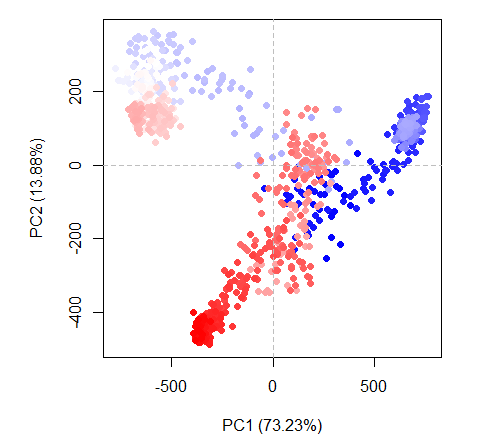


**Fig. S8.** Principal Component Analysis performed on all systems within three replicates of 300 ns MD simulations. The duration of the simulation can be seen in the color spectrum changing from blue to white to red. Blue, white, and red respectively represent the initial, middle, and end of the trajectory time.


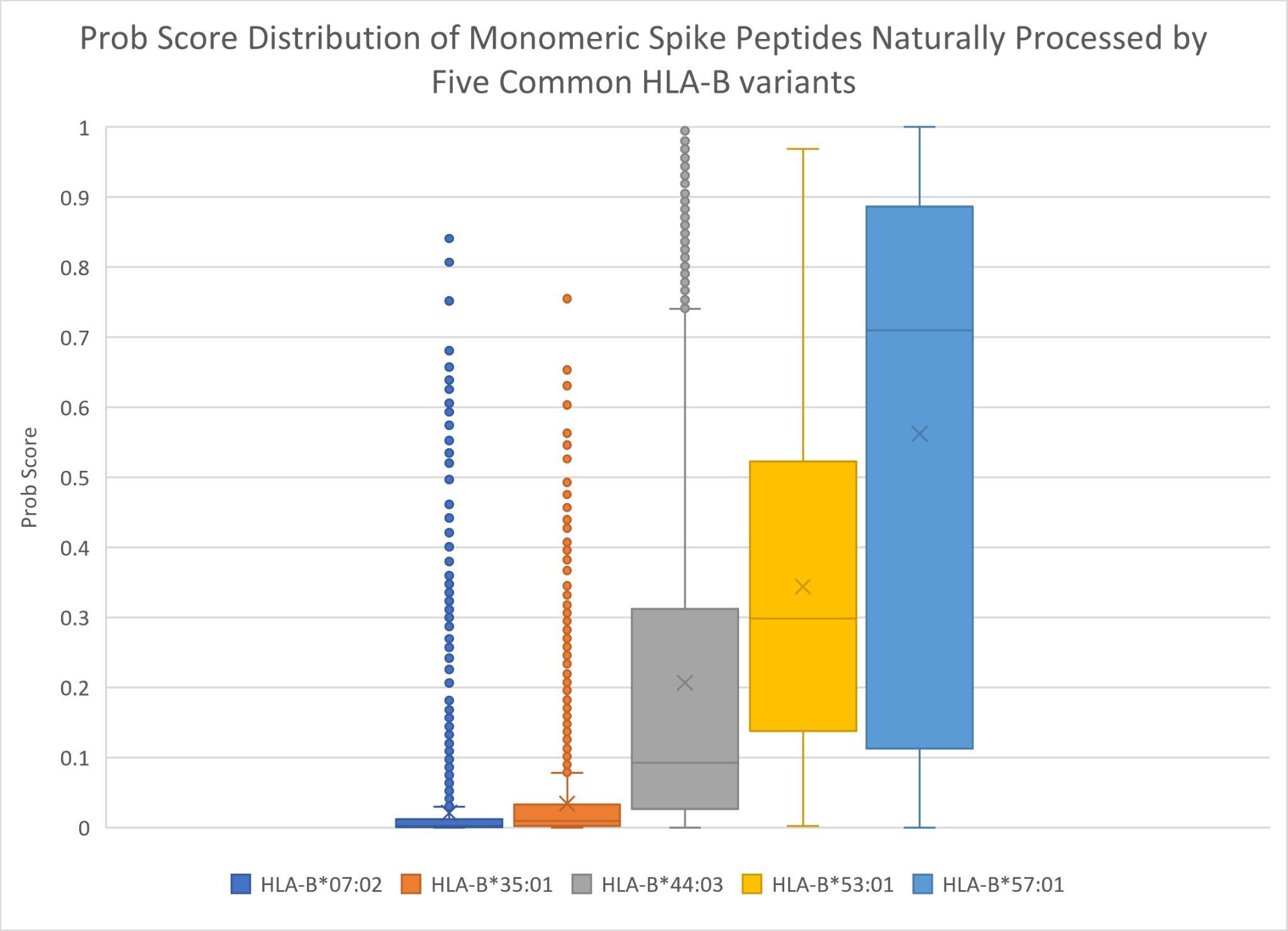


**Fig. S9.** Prob score distribution of monomeric spike peptides naturally processed by five common HLA-B variants. Prob scores depict the binding affinity of peptides to each HLA-B variant, providing insight into the probability of peptide binding.


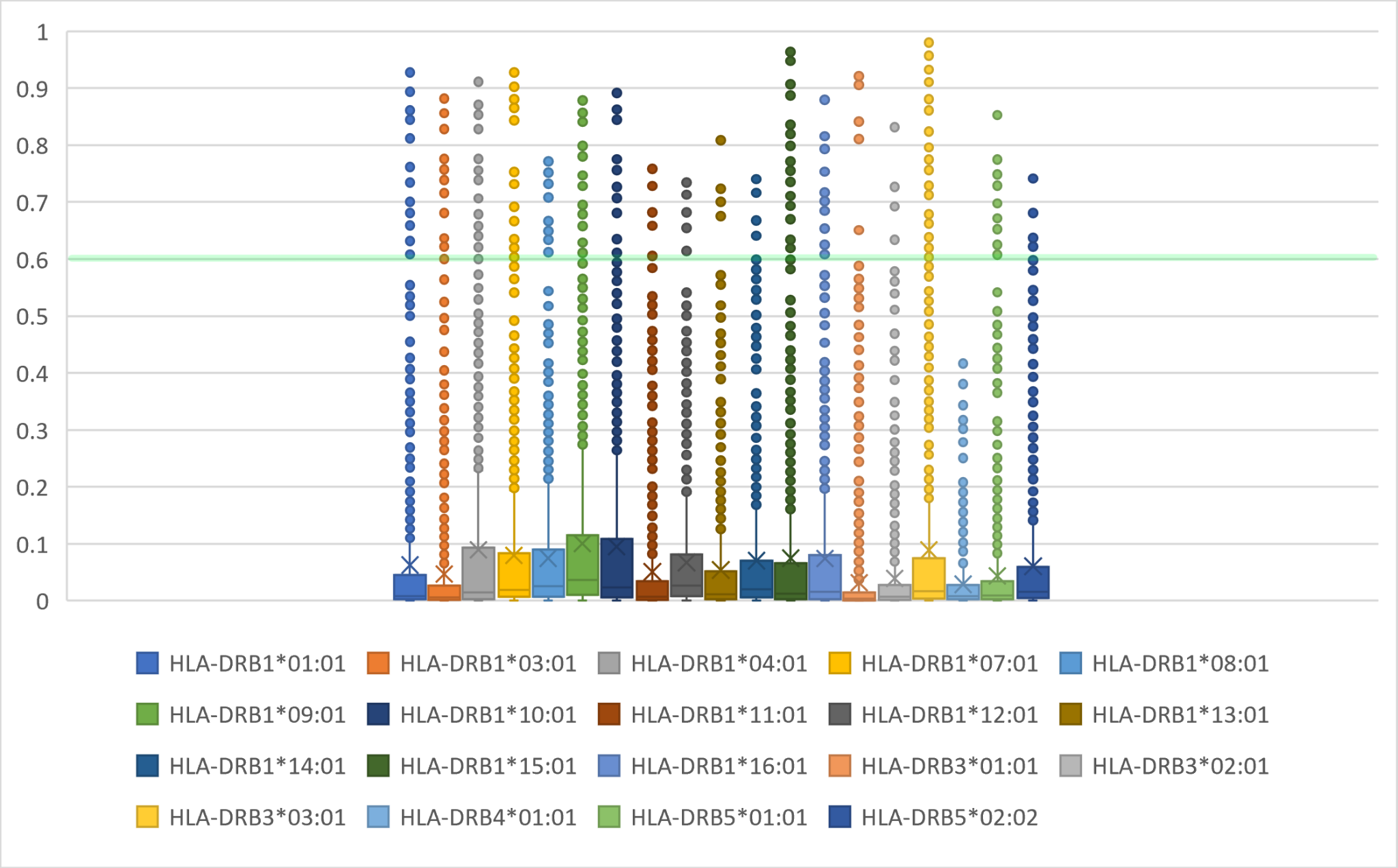


**Fig. S10.** MHC-II binding predictions were performed across the 19 most common HLA-DR alleles.
